# Transcriptome Analysis Identifies Candidate Genes Related to Triacylglycerol and Pigment Biosynthesis and Photoperiodic Flowering in the Ornamental and Oil-Producing Plant, *Camellia reticulata* (Theaceae)

**DOI:** 10.3389/fpls.2016.00163

**Published:** 2016-02-23

**Authors:** Qiu-Yang Yao, Hui Huang, Yan Tong, En-Hua Xia, Li-Zhi Gao

**Affiliations:** ^1^Plant Germplasm and Genomics Center, Germplasm Bank of Wild Species in Southwest China, Kunming Institute of Botany, Chinese Academy of SciencesKunming, China; ^2^University of Chinese Academy of SciencesBeijing, China

**Keywords:** *Camellia reticulata*, transcriptome, triacylglycerol biosynthesis, photoperiodic flowering pathway, pigment biosynthesis, EST-SSRs

## Abstract

*Camellia reticulata*, which is native to Southwest China, is famous for its ornamental flowers and high-quality seed oil. However, the lack of genomic information for this species has largely hampered our understanding of its key pathways related to oil production, photoperiodic flowering process and pigment biosynthesis. Here, we first sequenced and characterized the transcriptome of a diploid *C. reticulata* in an attempt to identify genes potentially involved in triacylglycerol biosynthesis (TAGBS), photoperiodic flowering, flavonoid biosynthesis (FlaBS), carotenoid biosynthesis (CrtBS) pathways. *De novo* assembly of the transcriptome provided a catalog of 141,460 unigenes with a total length of ~96.1 million nucleotides (Mnt) and an N50 of 1080 nt. Of them, 22,229 unigenes were defined as differentially expressed genes (DEGs) across five sequenced tissues. A large number of annotated genes in *C. reticulata* were found to have been duplicated, and differential expression patterns of these duplicated genes were commonly observed across tissues, such as the differential expression of *SOC1_a, SOC1_b*, and *SOC1_c* in the photoperiodic flowering pathway. Up-regulation of *SAD_a* and *FATA* genes and down-regulation of *FAD2_a* gene in the TAGBS pathway in seeds may be relevant to the ratio of monounsaturated fatty acid (MUFAs) to polyunsaturated fatty acid (PUFAs) in seed oil. *MYBF1*, a transcription regulator gene of the FlaBS pathway, was found with great sequence variation and alteration of expression patterns, probably resulting in functionally evolutionary differentiation in *C. reticulata*. *MYBA1_a* and some anthocyanin-specific biosynthetic genes in the FlaBS pathway were highly expressed in both flower buds and flowers, suggesting important roles of anthocyanin biosynthesis in flower development. Besides, a total of 40,823 expressed sequence tag simple sequence repeats (EST-SSRs) were identified in the *C. reticulata* transcriptome, providing valuable marker resources for further basic and applied researches on this economically important *Camellia* plant.

## Introduction

*Camellia reticulata* Lindley, one of the most famous plants in the family Theaceae (Huang et al., 2013), is an evergreen flowering tree or shrub naturally distributed in Southwest China (Ming et al., 2000). It primarily occurs in mountainous regions with poor soil conditions and a long dry season due to the subtropical plateau monsoon climates. Natural populations with different polyploidy levels (i.e., 2*n* = 2*x*, 4*x*, 6*x*; *x* = 15) of *C. reticulata* resulted from natural hybridization and polyploidization were found across Southwest China (Liu and Gu, 2011), which form a greatly valuable gene pool to be explored for *Camellia* breeding programs in the future. Compared with the common camellia oil plant, *C. oleifera, C. reticulata* is very welcome in this region because of its higher economic returns especially in terms of seed size, seed yield and oil content (Liu and Ma, 2010). It is well recognized that the oil extracted from *Camellia* seeds is similar to that from well-known olive in FA composition, making it a valuable edible oil source for daily consumption. Indeed, the camellia oil contains a high content of unsaturated FAs (UFAs), comprising 60–80% oleic acid and 5–10% linoleic acid (Liu and Ma, 2010; Ma et al., 2011) and nearly undetectable content of very long-chain FAs (VLCFA, >C_20_) (Ma et al., 2011). The ratio of monounsaturated FAs (MUFAs) to saturated FAs (SFAs) in the camellia oil is close to the optimal ratio following the Somopoulos' “Omega Diet” (Simopoulos and Robinson, 1999). These features are considered having balanced and healthy effects in reducing the risk of obesity, cancer, and heart disease. Researches in the rapeseed and *Arabidopsis* indicated that genes involved in triacylglycerol (TAG) biosynthesis (TAGBS) pathway are very much related to the oil composition and yield (Baud and Lepiniec, 2010). The TAGBS pathway mainly includes two conceptually simplified systems: the biosynthesis and modification of fatty acids (FAs), and TAG assembly (Baud and Lepiniec, 2010; Xu et al., 2011). FAs are *de novo* synthesized in the plastids with acetyl-CoA as a common precursor, and exported toward the cytosolic compartment as FA-CoA esters, which mainly contains FAs with double bonds of less than two and carbons of no longer than 18. FA modification generates a variety of FAs and involves enzymes contributing to FA elongation (>C_20_) and polyunsaturated FA (PUFA) biosynthesis, such as BETA-KETOACYL -[ACYL-CARRIER PROTEIN] REDUCTASE (KAR) (Slabas et al., 1992), and BETA-KETOACYL-COA SYNTHASE (KCS) (Joubes et al., 2008), FATTY-ACID DESATURASE (FAD) (Okuley et al., 1994; Ma and Browse, 2006), and PHOSPHATIDYLCHOLINE:DIACYLGLYCEROL CHOLINEPHOSPHOTRANSFERASE (PDCT) (Lu et al., 2009). The TAG assembly occurs in the endoplasmic reticulum and involves four consecutive enzymatic reactions catalyzed by GLYCEROL-3-PHOSPHATE ACYLTRANSFERASE (GPAT), LYSOPHOSPHOLIPID ACYLTRANSFERASE (LPAT), PHOSPHATIDIC ACID PHOSPHATASE (PAP), and DIACYLGLYCEROL ACYLTRANSFERASE (DGAT) (Baud and Lepiniec, 2010). Although the majority of candidate genes in TAGBS pathway were identified in the *C. oleifera* transcriptome using the 454 sequencing platform (Xia et al., 2014), their expression patterns are still largely unexplored in other *Camellia* species such as *C. reticulata*. The regulation of unique FA composition of camellia oil requires to be further studied, which will generate useful information to guide *Camellia* improvement programs.

*C. reticulata* is also a well-known camellia flower, which has been cultivated as a popular gardening plant in its indigenous region for at least 1300 years (Yu and Bruce, 1980). This ornamental woody plant is notable for large flowers, brilliant colors, various cultivars, and long florescence (Yu and Bruce, 1980; Liu and Gu, 2011). Extensive research efforts have advanced our knowledge regarding the genetic basis of photoperiodic flowering process in higher plants, particularly in *A. thaliana* for example. The photoperiodic flowering pathway overlaps with the circadian rhythm network, which acts as an endogenous timing system, enabling plants to promote flowering in response to photoperiod (Mas, 2005; Andres and Coupland, 2012). The photoperiod and irradiance are mainly perceived by mature leaves and mediated by many genes, mainly including *FLAVIN-BINDING KELCH REPEAT F BOX PROTEIN* (*FKF1*) (Imaizumi et al., 2005), *GIGANTEA* (*GI*) (Fowler et al., 1999), and *CONSTANS* (*CO*) (Putterill et al., 1995; Valverde et al., 2004). *CO* as the key regulator in the photoperiodic flowering pathway actively promotes the transcription of *FLOWERING LOCUS T* (*FT*) gene under long-day conditions (Putterill et al., 1995; Valverde et al., 2004). The FT protein, also known as florigen, is believed to move to the shoot apical meristem where the flowering process occurs (Amasino and Michaels, 2010). It has been proposed that FT interacts with FLOWERING LOCUS D (FD) to form a FT-FD complex in the meristem (Wigge et al., 2005). The FT-FD complex promotes the transcription activation of *SUPPRESSOR OF OVEREXPRESSION OF CONSTANS* (*SOC1*) and a floral meristem-identity gene *AP1APETALA1* (*AP1*) (Putterill et al., 1995; Wigge et al., 2005). *SOC1* encodes a MADS box transcription factor that activates another floral meristem identity gene *LEAFY* (*LFY*) (Putterill et al., 1995; Samach et al., 2000). *LFY* and *APETALA1* (*AP1*) subsequently induce flower development at the anlagen of shoot apical meristem according to the ABC model (Jack, 2004; Andres and Coupland, 2012). A recent transcriptome sequencing and analysis of the summer-flowering *C. azalea* has provided insights into its floral bud development, which characterized some genes involved in the photoperiodic flowering pathway (Fan et al., 2015). More information about this pathway from other *Camellia* plants will facilitate horticulturists to breed new cultivars with a wide-range blooming periods.

Flavonoids and carotenoids, two major groups of colorful pigments, are supposed to be involved in flower coloration in *Camellia* species (Nishimoto et al., 2004; Li et al., 2009). The pathways of flavonoid biosynthesis (FlaBS) and carotenoids biosynthesis (CrtBS) have been clearly uncovered in *A. thaliana* and some other plant species (Cunningham and Gantt, 1998; Winkel-Shirley, 2001; Cazzonelli and Pogson, 2010). Flavonoids are derived from phenylalanine. For the first steps, PHENYLALANINE AMMONIA-LYASE (PAL) catalyzes the phenylalanine into cinnamate, and subsequently, CINNAMATE 4-HYDROXYLASE (C4L) and 4-COUMARATE-COA LIGASE (4CL) catalyze the conversion of cinnamate to p-coumaroyl-CoA. Then, CHALCONE SYNTHASE (CHS) as a rate-limiting enzyme converts the p-coumaroyl-CoA into chalcone (Dao et al., 2011). Flavones, flavonols, anthocyanins and proanthocyanidins (PAs) are synthesized from the common chalcone precursor along the FlaBS pathway. Evidences indicated that FlaBS pathway genes are largely regulated at the transcription level by a complex of transcription factors (TFs) including R2R3 MYB TFs (Czemmel et al., 2012) and basic helix–loop–helix (bHLH) TFs (Nesi et al., 2000). For example, the MYBA1 in *Vitis vinifera* is a R2R3 MYB TF specifically controlling the expression of *UFGT*, which encodes an enzyme responsible for the anthocyanin biosynthesis and able to determine the grape berry colors (Walker et al., 2007). Carotenoids are a group of isoprenoid molecules with characteristic color in the yellow to red range (Cazzonelli and Pogson, 2010). Carotenoids are synthesized from the five-carbon building blocks isopentenyl diphosphate (IPP) and its double-bond isomer dimethylallyl diphosphate (DMAPP), both of which are produced by the plastid-localized methylerythritol phosphate pathway (Phillips et al., 2008). Three IPP molecules are added to DMAPP by GERANYLGERANYL DIPHOSPHATE SYNTHASE (GGPS) to generate geranylgeranyl diphosphate (GGPP), a common precursor for the biosynthesis of carotenoids and several other groups of plastidic isoprenoids (Hirschberg, 2001). Although genes involved in these pathways have been extensively studied in other plants, more studies on these networks are needed in *Camellia* species. Transcriptome sequencing uncovered the candidate genes in the FlaBS pathway in *Camellia*, such as *C. sinensis* (Shi et al., 2011), *C. taliensis* (Zhang et al., 2015), and *C. chekiangoleosa* (Wang et al., 2014). However, there is still a lack of comprehensive expression profiling of the FlaBS genes. In particular, there is so far no information for the CrtBS pathway in *Camellia* plants.

The economic importance of *Camellia* species is largely due to the demand for young leaves (e.g., tea leaves from *C. sinensis*), ornamental flowers, and seed oil. Genes that are involved in TAGBS, FlaBS and CrtBS pathways and photoperiodic flowering are supposed to be highly relevant to the above-mentioned agricultural and horticultural traits. While comprehensive analysis on gene networks related to photoperiodic flowering and CrtBS is so far absent in *Camellia* plants, genes involved in TAGBS and FlaBS were reported in *C. sinensis* (Shi et al., 2011), *C. chekiangoleosa* (Wang et al., 2014), and *C. oleifera* (Xia et al., 2014). But, they are still far away from being well characterized in *Camellia* because of scarce information of gene expression profiles. Indeed, although RNA sequencing (RNA-Seq) datasets have been reported in several *Camellia* species, large-scale gene expression profiles were only available in *C. sinensis* to identify genes activated during cold acclimation (Wang et al., 2013), because other studies are unable to provide a broad expression analysis because of the limitation of sequencing strategies, such as 454 sequencing (Wu et al., 2013; Wang et al., 2014; Xia et al., 2014) or Illumina sequencing with a single library (Shi et al., 2011). Compared with the other *Camellia* species, very few genomic resources are available for *C. reticulata*. Nevertheless, a full catalog of genes and their expression profiles in *C. reticulata* are needed to better understand the above mentioned biological pathways.

Here, Illumina RNA-Seq was employed to *de novo* sequence the transcriptome of the diploid *C. reticulata*. The transcriptomes from the five tissues under normal development conditions were separately sequenced and compared to present a global survey of expression profiles as well as differential expression analysis in *C. reticulata*. A large set of unigenes was obtained to identify the majority of genes related to TAGBS, FlaBS, CrtBS, and photoperiodic flowering pathways. The characterization of EST-SSRs (expressed sequence tag, simple sequence repeat) from the transcriptome of *C. reticulata* has expanded valuable marker resources in breeding programs of the camellia community.

## Materials and methods

### Plant material, RNA isolation and illumina sequencing

The wild *C. reticulata* plant YB1-2, which was previously characterized as a diploid (Xia et al., 1994; Liu and Gu, 2011; Huang et al., 2013), was collected from Sichuan Province, China (Supplementary Table S1). A total of five tissues, including leaf buds, mature leaves, flower buds, flowers and immature fruits, were used for transcriptome sequencing and quantitative reverse transcription polymerase chain reaction (qRT-PCR) validation; blackening seeds were only used for qRT-PCR (Supplementary Figure S1). Samples were immediately frozen in liquid nitrogen and stored at −80°C. Total RNA of each sample was isolated using a modified CTAB (cetyl trimethylammonium bromide) method (Li L. et al., 2008). RNA samples were treated with RNase-free DNase I (Takara) to avoid DNA contamination. RNA quality was assessed using an Agilent 2100 BioAnalyzer.

For each RNA sample, Poly-A mRNA was enriched and then used to prepare a 350-bp paired-end cDNA library (2 × 100 nt) according to the Illumina protocol. Paired-end sequencing was performed on the Illumina HiSeq™ 2000 platform.

### Sequence data processing and *de novo* assembly

FastQC (http://www.bioinformatics.babraham.ac.uk/projects/fastqc/) was used to evaluate and visualize the sequence quality before and after trimming process. The trimming process was performed with Trimmomatic (Lohse et al., 2012), sequentially removing adapters, the first seven bases at the 5′ end of the reads due to sequence bias (Hansen et al., 2010), low quality bases (phred score < 20), and short reads (< 50 nt). The clean reads were *de novo* assembled using Trinity software (Grabherr et al., 2011), with parameter settings of “–group_pairs_distance 450 –min_contig_length 200 –CPU 4” and default options otherwise.

### Post-assembly processing

Three rounds of filtering process were performed to construct a high-quality nuclear transcriptome assembly. First, putative protein-coding sequences most likely from plant epiphytes and pathogens were identified and discarded using a previously described taxonomy-based method (Krasileva et al., 2013). Next, a second round of filtering process was performed using DeconSeq (Schmieder and Edwards, 2011) against a local database constructed from genomic sequences of candidate contaminant species (i.e., human, bacteria, virus, fungi, and top 20 epiphyte or pathogen species inferred from the taxonomy-based filtering step). Finally, mitochondrial and plastidial transcripts were identified and removed by searching against publicly available mitochondrion and plastid genomes of dicotyledonous plants.

Then the filtered sequences were subjected to cluster analysis using CD-HIT-EST (Huang et al., 2010) with the following parameters: -r 1 and -c 0.9. The longest sequence of each cluster was used as a representative sequence (i.e., unigene).

### Sequence annotation

Homology searches were performed against the UniProtKB/Swiss-Prot (Swiss-Prot), UniProtKB/TrEMBL (TrEMBL), NCBI (National Center for Biotechnology Information) non-redundant protein database (NR), *A. thaliana* proteins database (TAIR10), and *V. vinifera* proteins database (IGGP 12X) using a BLASTx procedure with an *e*-value threshold of 10^−3^. For each unigene, the best hit against these databases was used to predict sequence orientation, coding DNA sequence (CDS), and polypeptide sequence with GeneWise software (Birney and Durbin, 2000). The predicted peptide sequences were analyzed to assign corresponding protein domains and families by using the pfam_scan tool against the Pfam database (Punta et al., 2012). Gene ontology (GO) were obtained based on the NR database annotations using Blast2GO software (version 2.6.5) (Conesa et al., 2005), and then WEGO software (Ye et al., 2006) was used to obtain GO functional classifications. The unigenes were also submitted to the online KEGG (Kyoto Encyclopedia of Genes and Genomes) Automatic Annotation Server (KAAS) to obtain enzyme commission (EC) numbers and associated KEGG orthology (KO) identifiers that are directly linked to objects in the KEGG pathway map (Moriya et al., 2007). The KAAS annotation was performed with single-directional best hit method using angiosperm species data sets as reference. We focused on unigenes assigned to four pathways: photoperiodic flowering, TAGBS, FlaBS, and CrtBS. Finally, we performed a manual curation for these unigenes.

### Transcript abundance and expression-based analysis

Paired end reads were aligned to the assembly by Bowtie (Langmead and Salzberg, 2012), and the resulting alignments were used to estimate expression abundances in FPKM (expected number of fragments per kilobase of transcript sequence per millions base pairs sequenced) by RSEM (Li and Dewey, 2011). The Bioconductor tool edgeR (Robinson et al., 2010) was employed to calculate the expression abundance fold change based on pairwise comparisons of normalized FPKM among five sequenced tissues using exact statistical method with default settings. Differentially expressed genes (DEGs) were defined with a threshold of fold change ≥ 4 and false discovery rate (FDR) ≤ 0.001. Enrichment analyses of DE gene sets in KEGG pathways or the GO database were performed using the online tool KOBAS (KEGG Orthology Based Annotation System, http://kobas.cbi.pku.edu.cn/) (Xie et al., 2011) with the expressed genes (FPKM ≥ 1) as background (corrected *p* ≤ 0.05).

### qRT-PCR analysis

Primers used for qRT-PCR assays were designed with the Primer Express software (version 3.0, Applied Biosystems), and they amplified PCR products varied from 80 to 159 bp (Supplementary Table S2). The housekeeping gene *ELONGATION FACTOR 1 ALPHA* (*EF1A*) was used as internal reference control for normalization (Nicot et al., 2005). qRT-PCR assays were performed as described previously (Xu et al., 2011). The relative expression of the genes was calculated using the 2^−ΔΔCt^ method.

### EST-SSR identification and polymorphism survey

To increase confidence, only unigenes with length ≥500 nt were used for EST-SSR identification. They were searched by using the MISA program (Thiel et al., 2003), mining EST-SSRs with 2- to 6-nt motifs and a minimum length of 12 nt.

Twenty EST-SSRs were chosen for polymorphism survey in 24 individual *C. reticulata* plants (Supplementary Tables S1, S3). PCR primer pairs were designed using Invitrogen Vector NTI (version 10) with standard criteria. DNA extraction was performed as described previously (Doyle and Doyle, 1987). Standard PCR amplifications and electrophoresis were performed as described previously (Tong et al., 2013). The number of alleles per locus (NA), effective number of alleles (NE), Shannon's diversity index (I), observed heterozygosity (Ho), and expected heterozygosity (He) were estimated with POPGENE software (version 1.32) (Yeh et al., 1999). The polymorphic information content (PIC) value was calculated according to a previously described formula (Botstein et al., 1980).

## Results and discussion

### Sequencing, *de novo* assembly and assessment of the *C. reticulata* transcriptome

**Figure 1:**
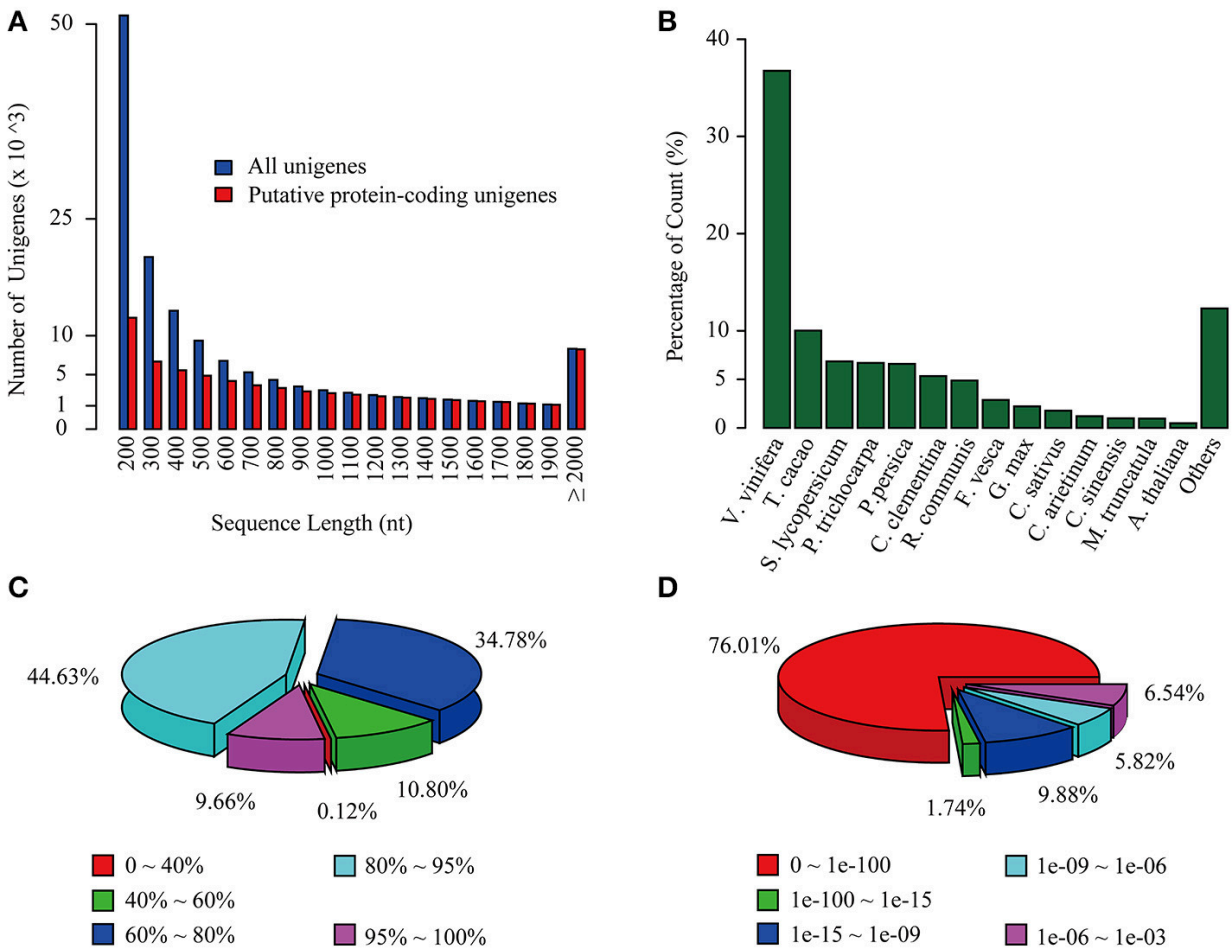
**Overview of *C. reticulata* transcriptome assembly and its homology search against the NR database**. **(A)** Length distribution of total unigenes (blue) and putative protein-coding unigenes (red). **(B)** Species, **(C)** similarity, and **(D)** *E*-value distributions of the top BLAST hits for each unigene against the NR database.

Distinct cDNA libraries of leaf buds, mature leaves, flower buds, flowers, and immature fruits (Supplementary Figure S1) from a wild diploid *C. reticulata* plant were separately sequenced with Illumina HiSeq™ 2000, generating a total of 394.9 million 100 nt paired-end raw reads. After a stringent trimming process, about 311.3 million clean reads (78.8% of total raw data) with 26.2 billion nucleotides in total were retained (Table 1). The high-quality reads were *de novo* assembled into contigs with Trinity, which has been shown to be the best single k-mer assembler for *de novo* assembly from RNA-Seq short reads (Grabherr et al., 2011; Zhao et al., 2011). As direct output from the assembly procedure often includes contaminants and redundant sequences, assembled sequences were subjected to do a post-assembly processing to obtain a high-quality nuclear transcriptome (see Material and methods). As a result, an assembly of 232,428 contigs with a total size ~186.4 million nucleotides (Mnt) was established for *C. reticulata*. After clustering analysis, 141,460 unigenes with a total size ~96.1 Mnt were generated (Table 1). The unigenes ranged from 200 to 9,880 nt, with a mean length of 679 nt and an N50 length of 1080 nt (Table 1 and Figure 1A).

**Table 1:** **Summary of the sequencing data and transcriptome assembly of *C. reticulata***.

	**Raw reads**	**Clean reads**	**Retain rate (%)**	**Clean nucleotides (nt)**
Leaf buds	75,791,012	57,655,612	76.1	4,700,656,880
Mature leaves	95,982,836	74,474,740	77.6	6,113,913,256
Flower buds	70,418,086	57,626,233	81.8	4,929,278,585
Flowers	71,987,410	59,351,076	82.4	5,114,541,561
Immature fruits	80,714,310	62,182,007	77.0	5,323,094,674
Total	394,893,654	311,289,668	78.8	26,181,484,956
	**Contigs**	**Unigenes**		
Total number	232,428	141,460		
Total length (nt)	186,434,209	96,117,212		
Mean length (nt)	802	679		
N50 (nt)	1,255	1,080		
GC content (%)	42.4	41.8		
Number of length ≥ 500 nt	119,933	57,162		
Number of length ≥ 1000 nt	63,845	27,880		
Reads mapping rate (%)	84.5	80.8		

Though a systematic evaluation standard is not yet available for transcriptome assembly of non-model organisms (Martin and Wang, 2011), we tried to overview the quality of our assembly with several broadly used parameters and by comparing it to independent databases from closely related species. Reads mapping showed that the proportion of reads assembled was 84.5% (Table 1), which is a comparable alignment rate to that of other *de novo* assemblies. 86.3% of the mapped paired-end reads aligned concordantly, showing good physical evidence of sequence contiguity. The unigenes was compared to available ESTs and protein sequences of *Camellia* plants from NCBI. Of 111,905 non-redundant *Camellia* ESTs, 96,092 (85.9%) ESTs were represented in our assembly (Megablast, *E* = 10^−9^), among which 72,924 (75.9%) ESTs were matched with more than 80% identity and 80% coverage (Supplementary Figure S2). Of 256 non-redundant *Camellia* proteins (≥300 AA), 255 (99.6%) matched to at least one assembled unigene (BLASTx, *E* = 10^−9^), among which 215 (84.3%) were represented with ≥80% coverage. These results demonstrated that the assembly successfully constructed a large number of homologous transcripts with desirable lengths. Furthermore, chimeric assembly level was inspected using the top longest assembled sequences (Van Belleghem et al., 2012). All of the 10 longest unigenes (≥7400 nt) in our assembly positively matched with long gene sequences in public databases without obvious chimeric evidence, and 9 of them were confirmed to have good alignment confidence (70% identity and 80% coverage) at both the cDNA and protein levels (Supplementary Table S4). These assessments indicate that the *C. reticulata* transcriptome constructed herein possessed desirable completeness, accuracy, and contiguity.

To facilitate the access and utilization of the *C. reticulata* transcriptome data, the sequences of raw reads and unigenes were deposited in the NCBI (BioProject ID PRJNA297756). The full list of transcript sequences is available upon request.

### Functional annotation and gene ontology classification

To annotate the transcriptome with putative functions, similarity searches for each unigene were performed using BLASTx against five public databases: Swiss-Prot, TrEMBL, NR, TAIR10 and the *Vitis vinifera* protein database. With an *E*-value threshold of 10^−3^, 69,922 (49.4% of the total) unigenes were significantly matched with known proteins in at least one of the searched databases (Table 2 and Supplementary Table S5), and 42,042 (29.7% of the total) unigenes had significant hits with proteins presented in all of the five databases (Table 2). All of the 69,922 unigenes matched to known proteins were considered as putative protein-coding sequences. The length distribution of these putative protein-coding sequences is shown in Figure 1A, showing that the longer sequence would have a higher probability of being matched.

**Table 2:** **Summary of functional annotation for *C. reticulata* unigenes**.

**Annotation**	**Number of unigenes**	**Percentage of matched unigenes (%)**	**Number of unique homologs**
Total unigenes	141,460		
Annotated unigenes	69,922	49.4	
**BLASTx AGAINST PUBLIC PROTEIN DATABASES**			
NR	68,026	48.1	41,413
TrEMBL	62,618	44.3	37,374
Swiss-Prot	49,469	35.0	16,373
*A. thaliana* proteins (TAIR10)	56,664	40.1	17,368
*V. vinifera* proteins	59,968	42.4	15,763
Unigenes matched at least one database	69,922	49.4	
Unigenes matched all five databases	42,042	29.7	
**DOMAIN/GO ANNOTATION**			
Search against PFAM database	47,693	33.7	
Annotated with GO terms	39,301	27.8	

For the NR annotations, the top-hit species distribution is shown in Figure 1B. We found that 36.75% of the mapped unigenes had the highest matches to genes from *V. vinifera*, followed by *Theobroma cacao* (10.02%) and *Solanum lycopersicum* (6.85%) (Figure 1B). Overall, 54.29% of the matched sequences were mapped with similarity >80% (Figure 1C), and 76.01% of the matched sequences showed strong homology with an *E* < 10^−100^ (Figure 1D). Notably, the annotated genes of *Aribidopsis* and *V. vinifera* on average have more than three counterparts in *C. reticulata* transcriptome (17,368 vs. 56,664 and 15,763 vs. 59,968, respectively, see Table 2), suggesting that the hypothesis of genome duplication events in *C. sinensis* probably might occur in *C. reticulata* (Shi et al., 2011).

Sequence orientation, CDS, and polypeptide sequence were predicted using GeneWise software (Birney and Durbin, 2000). For each putative polypeptide sequence, its functional domain and associated family was inferred by pfam_scan analysis against the Pfam database. The top 10 abundant families and domains are listed in Supplementary Table S6. The “pentatricopeptide repeat” (PF01535), which is thought to be involved in organelle biogenesis (Lurin et al., 2004), was the most abundant protein family. The “protein kinase domain” (PF00069), which functions in a process called phosphorylation, was the most abundant protein domain.

The GO classification system that describes gene function into three major categories (biological processes, molecular functions, and cellular components) and additional subcategories was also applied to putative gene functions. Overall, 39,301 unigenes were assigned to 131,466 GO terms (3736 unique GO terms; Supplementary Table S7) that were further classified into 47 subcategories (Supplementary Figure S3). The most abundant GO subcategories for biological processes, molecular functions, and cellular components were metabolic process (GO:0008152), binding (GO:0005488), and cell (GO:0005623), respectively.

### KEGG pathway analysis

KEGG annotation and pathway assignment can help clarify the biological functions of genes in terms of networks (Moriya et al., 2007). A total of 21,940 unigenes were matched to 3,278 unique KO groups (Supplementary Table S7), and 6,803 unigenes were assigned with 769 unique EC numbers. Pathway mapping assigned 13,316 unigenes (2,116 KO groups) into 333 functional pathways (Supplementary Table S7). “Metabolic pathways” (ko01100) had the largest number of KO identifiers (794, 37.5%), followed by “biosynthesis of secondary metabolites” (352, 16.6%, ko01110), “microbial metabolism in diverse environments” (129, 6.1%, ko01120), “ribosome” (119, 5.6%, ko03010), and “spliceosome” (103, 4.9%, ko03040). The KEGG pathway annotation results provided valuable information that allowed us to relate genes to specific physical processes such as TAGBS, FlaBS, CrtBS, and photoperiodic flowering, which will be demonstrated in the following paragraphs.

### EST-SSR marker identification and polymorphism survey

57,162 unigenes with a length of at least 500 nt were used to identify EST-SSRs. As a result, a total of 40,823 EST-SSRs were detected in 25,188 unigenes (44.1% of the total searched unigenes; Table 3 and Supplementary Table S8), equivalent to an average frequency of one SSR per 1.74 kilo-nucleotides (Knt) of the transcriptome sequences. This result was slightly more frequent than those reported in a corresponding study on tea (2.41 Knt) that used different parameters (Tan et al., 2013). The most abundant repeat type was trinucleotide (14,688, 36.0%), followed by dinucleotide (13,837, 33.9%), and tetranucleotide (5,759, 14.1%). Out of 411 repeat motifs identified, the most frequent was AG/CT (11,367, 27.8%), followed by AAG/CTT (3,369, 8.3%), ACC/GGT (2,711, 6.6%), ATC/ATG (2,176, 5.3%), and AGG/CCT (1,646, 4.0%) (Supplementary Table S8).

**Table 3:** **EST-SSRs present in the *C. reticulata* transcriptome**.

**Repeat type**	**Number of repeat units**											
	**3**	**4**	**5**	**6**	**7**	**8**	**9**	**10**	**11**	**12**	**≥13**	**Total**
Dinucleotide	–	–	–	3650	2723	2807	2780	1511	353	13	4	13,841
Trinucleotide	–	8891	3246	1596	862	83	3	5	0	0	2	14,688
Tetranucleotide	4560	842	303	45	4	0	3	1	1	0	0	5759
Pentanucleotide	2215	594	57	5	0	0	0	0	0	0	0	2871
Hexanucleotide	2939	634	48	29	8	1	3	2	0	0	0	3664
Total												40,823

Out of 20 EST-SSR primer pairs chosen for polymorphism survey, 18 were successfully amplified in 24 *C. reticulata* accessions (Supplementary Tables S1, S3 and Supplementary Figure S4A). Among the 18 working primer pairs, 17 successfully amplified PCR products with the expected sizes, and one pair (SSR11) generated a larger PCR product (~270 bp) than expected size of 159 bp, suggesting that there may be a short intron within the amplicon. Our polymorphism survey showed that seven primers pairs could amplify polymorphism bands among the 24 *C. reticulata* representative samples (Supplementary Figures S4B–H). The PIC values varied from 0.33 to 0.69 with an average of 0.50, and the NA is 3.57 (Table 4). These results clearly demonstrated that EST sequences derived from RNA-Seq are effective and valuable resources to develop polymorphic SSR markers, which are essential in various applications such as population genetic structuring and linkage mapping.

**Table 4:** **Allelic diversity attributes of seven polymorphic EST-SSRs**.

	**N**	**NA**	**NE**	**PIC**	**Ho**	**He**	**I**
SSR1	24	4	2.40	0.41	0.71	0.58	1.01
SSR3	24	3	2.78	0.56	0.71	0.64	1.06
SSR11	23	3	2.38	0.54	0.87	0.58	0.94
SSR16	23	2	1.84	0.41	0.54	0.46	0.65
SSR17	24	4	2.19	0.33	1.00	0.54	0.89
SSR18	24	5	3.71	0.69	0.58	0.73	1.41
SSR19	24	4	2.59	0.55	0.92	0.61	1.06
Mean	24	3.57	2.56	0.50	0.76	0.59	1.00

N, The sample size.

### Transcriptome expression profiling and differential expression analysis

Expression levels of unigenes were determined by aligning the RNA-Seq reads from each library to the assembly. The default empirical abundance threshold of 1 FPKM was used to evaluate whether a gene was positively expressed (Vogel and Marcotte, 2012; Gonzalez-Porta et al., 2013). As a result, 94,450 unigenes were positively expressed in at least one of the libraries. Among them, 17,956 unigenes were expressed in all five tissues, and 8,837, 13,963, 3,265, 10,540, and 7,094 unigenes were specifically expressed in leaf buds, mature leaves, flower buds, flowers, and immature fruits, respectively (Supplementary Figure S5A). Based on expression levels, the sequence depths of expressed unigenes varied across a broad range. The top 1% of abundantly expressed unigenes accounted for 60% of the overall abundance value, while the top 10% abundantly expressed unigenes were 86% of the overall abundance value (Supplementary Figure S5B).

A total of 22,229 unigenes were defined as DEGs (fold change = 4 and FDR = 0.001); and 18,639 (83.85%) of these were putative protein-coding genes, indicating specific enrichment of functional genes in this dataset. All of the DEGs were assigned to five groups (DELB, DEML, DEFB, DEFL, and DEFR) according to the tissue identity where their highest expression occurred (Supplementary Table S9). As a result, Group DEML was assigned with the maximum number of DEGs (5,451), followed by Group DELB (5,073), Group DEFL (4,599), Group DEFR (3,756), and Group DEFB (3,350) (Supplementary Table S9). KEGG pathway or GO enrichment analysis of the DE unigenes in each group indicated that unigenes preferentially expressed in a tissue are highly related to the specific functions of that tissue (Supplementary Table S10). For example, Group DEML expressed most preferentially in mature leaves enriched genes involved in “photosynthesis,” “carbon fixation,” “fructose, mannose and nitrogen metabolism,” “porphyrin and chlorophyll metabolism,” as well as “carotenoid biosynthesis.” Group DEFB and DEFL showed preferential expression of genes involved in different functional processes, which are related to flower development in different stages (flower buds and flowers), such as “plant hormone signal transduction,” “pigment accumulation” (GO:0043476), “carotenoid biosynthesis” and “phenylpropanoid biosynthesis.” Genes from group DEFR were highly expressed in immature fruits, and included genes functioned in “flavonoid biosynthesis,” “phenylpropanoid biosynthesis,” “flavone and flavonol biosynthesis,” and “biosynthesis pathways of secondary metabolites.” Collectively, the DEGs are able to mirror physiological differences among the five tissues. These results will facilitate the identification of genes that function in specific physiological programs.

### qRT-PCR validation

**Figure 2:**
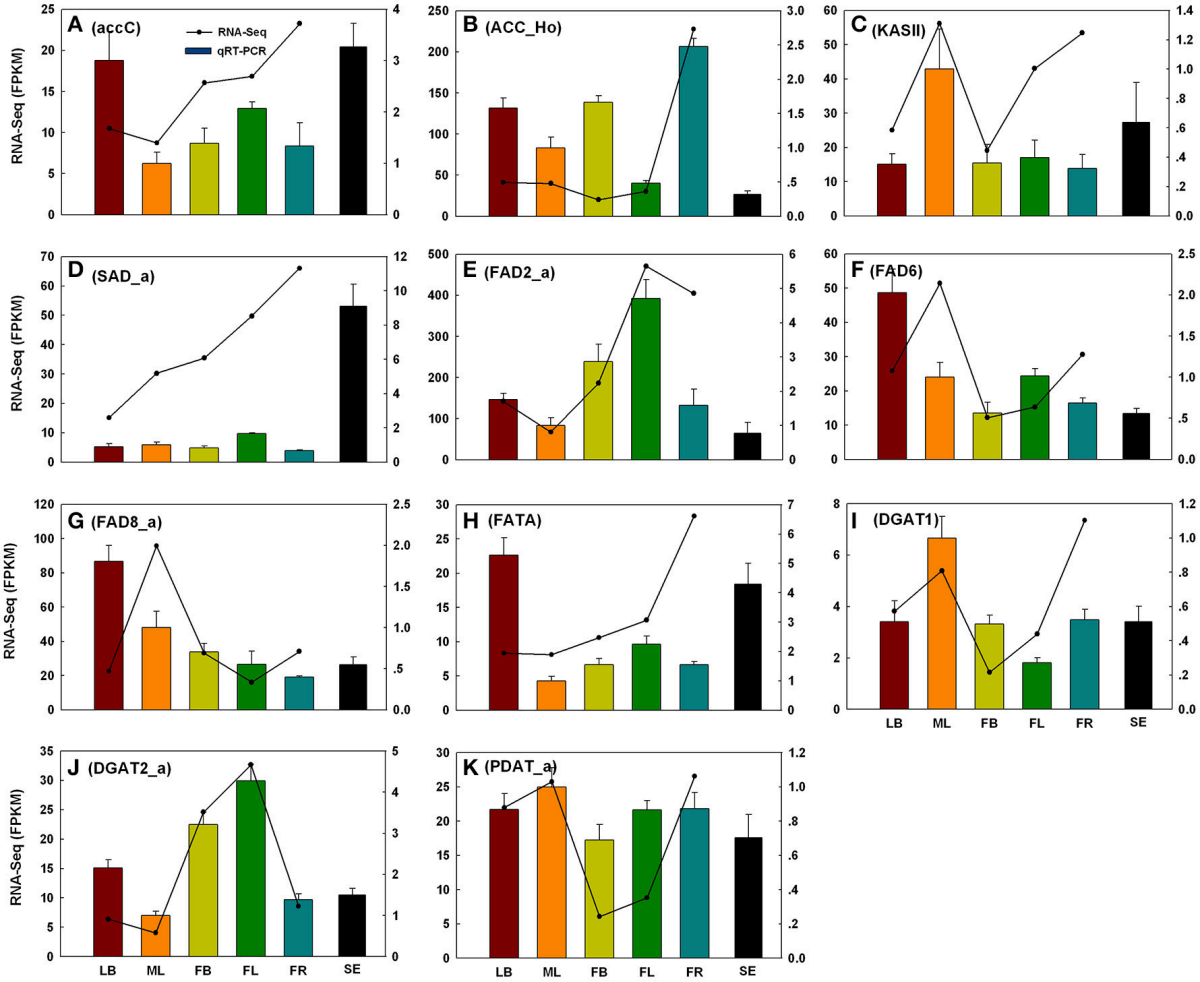
**qRT-PCR validations of 11 putative genes involved in TAG biosynthesis**. The histograms show the qRT-PCR results of accC **(A)**, ACC_Ho **(B)**, KAS II **(C)**, SAD_a **(D)**, FAD2_a **(E)**, FAD6 **(F)**, FAD8_a **(G)**, FATA **(H)**, DGAT1 **(I)**, DGAT2_a **(J)**, and PDAT_a **(K)**; the line charts show the FPKM values of these genes. qRT-PCR results represent the mean (±SD) of three biological replicates. Gene abbreviations can be referenced in Supplementary Table S12. LB, leaf buds; ML, mature leaves; FB, flower buds; FL, flowers; FR, immature fruits; SE, seeds.

**Figure 3:**
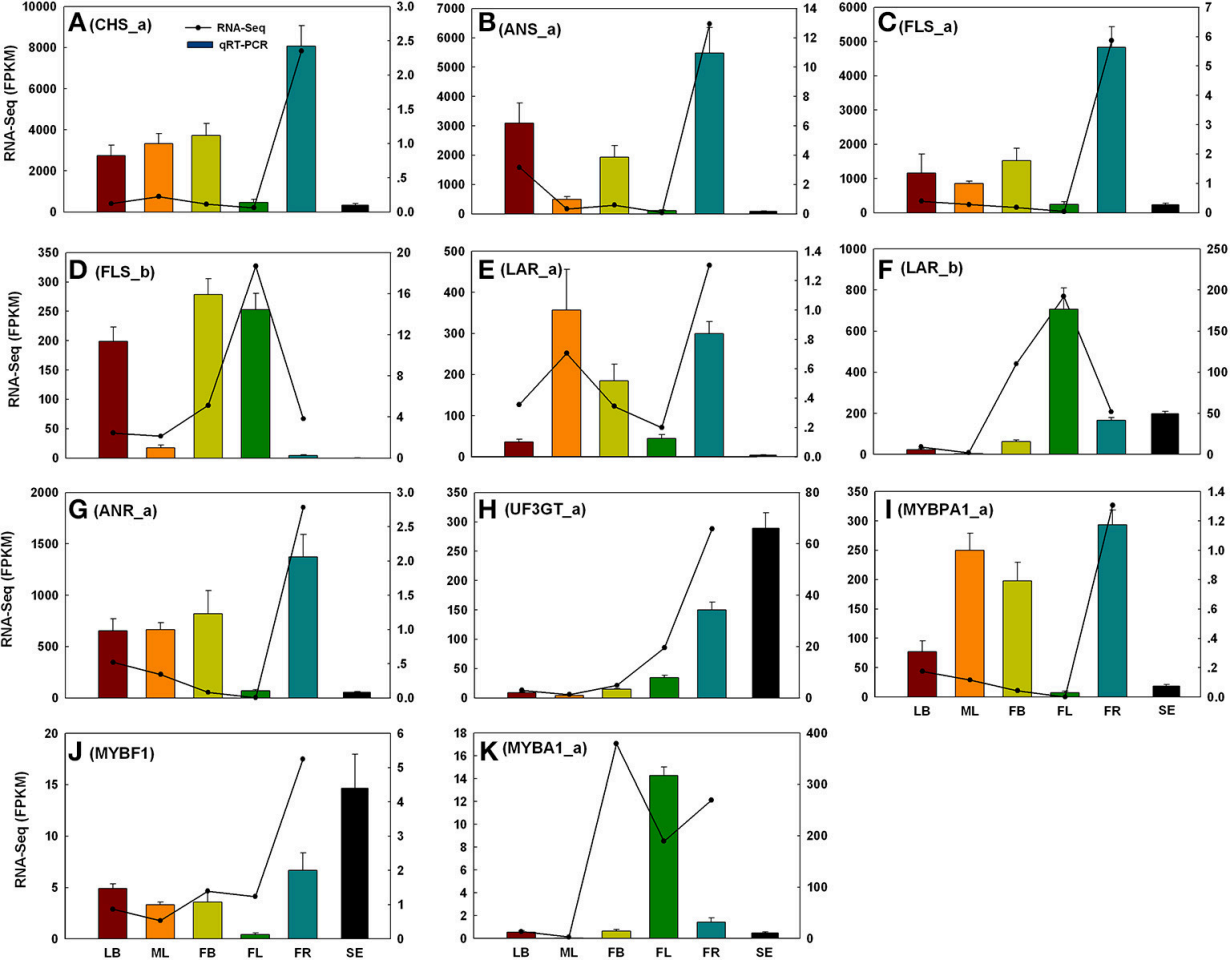
**qRT-PCR validations of 11 putative genes involved in flavonoid biosynthesis**. The histograms show the qRT-PCR results of CHS_a **(A)**, ANS_a **(B)**, FLS_a **(C)**, FLS_b **(D)**, LAR_a **(E)**, LAR_b **(F)**, ANR_a **(G)**, UF3GT_a **(H)**, MYBPA1_a **(I)**, MYBF1 **(J)**, and MYBA1_a **(K)**; the line charts show the FPKM values of these genes. qRT-PCR results represent the mean (± SD) of three biological replicates. Gene abbreviations can be referenced in Supplementary Table S13. LB, leaf buds; ML, mature leaves; FB, flower buds; FL, flowers; FR, immature fruits; SE, seeds.

To evaluate the reliability of RNA-Seq analysis, 22 gene homologs related to TAGBS and FlaBS pathways were selected for qRT-PCR test in six tissues (leaf buds, mature leaves, flower buds, flowers, immature fruits, and blackening seeds) (Supplementary Figure S1 and Supplementary Table S1). In general, our qRT-PCR results show a high degree of consistency with the RNA-Seq results (Figures 2, 3). Expression patterns of nine (40.9%) cases (Figures 2J,K, 3A–E,H,J) fit well with the RNA-Seq results across all five tissues. Ten (45.5%) cases (Figures 2B,C,E–G,I, 3F,G,I,K) had almost similar expression patterns but with very small partial inconsistences compared to the RNA-Seq results. It is rational and acceptable that there are certain differences in direct comparisons between the RNA-Seq and qRT-PCR results due to different normalization methods, bias in library preparation in RNAseq, and other technical biases (Bustin, 2002; Li et al., 2010; Zheng et al., 2011).

### Triacylglycerol biosynthesis pathway in *C. reticulata*

**Figure 4:**
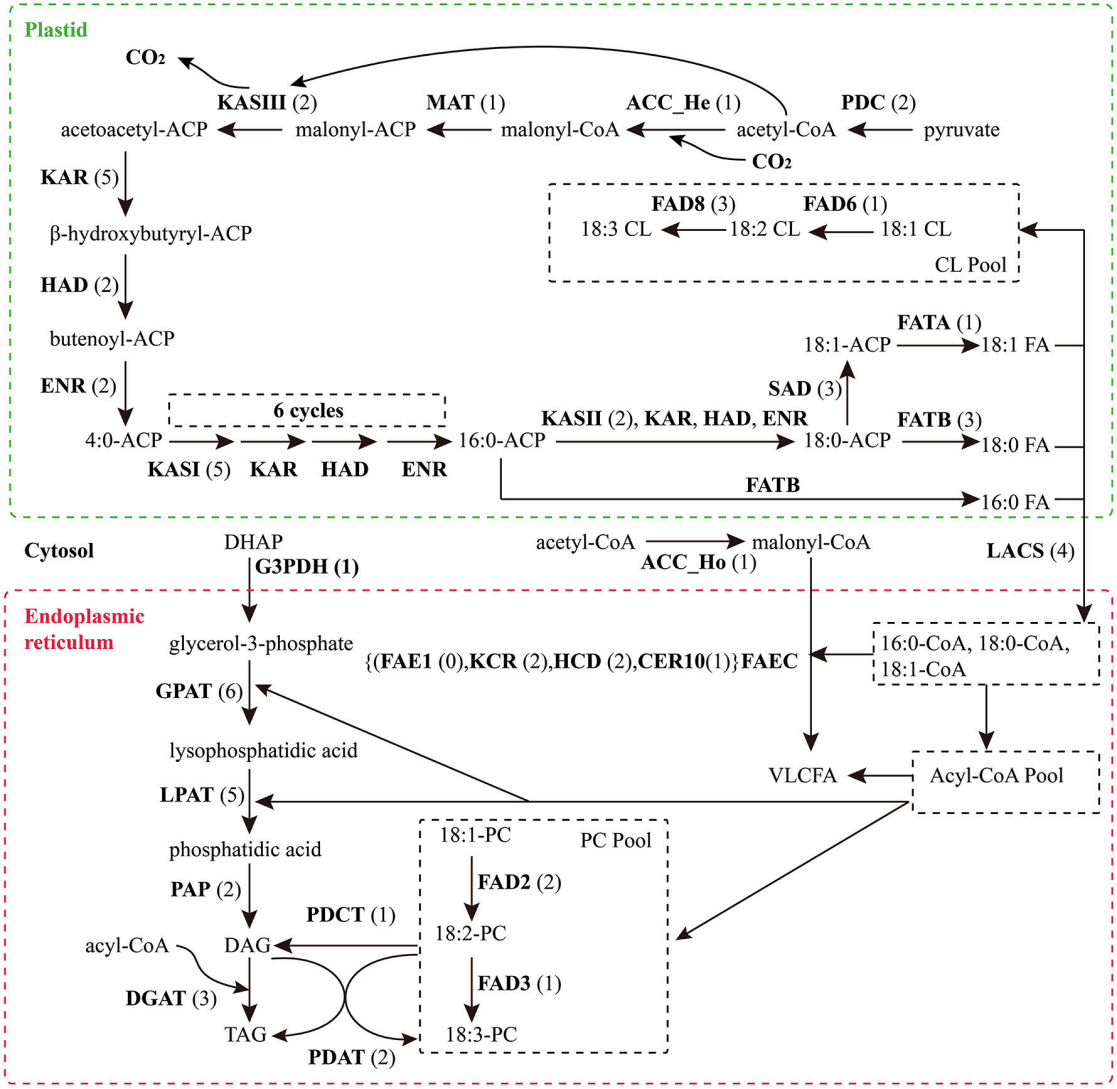
**Putative genes related to TAG biosynthesis in *C. reticulata***. The numbers in brackets following each gene name indicate the number of unigenes annotated to that gene. For each gene abbreviation, its full name and other information can be referenced in Supplementary Table S11.

Based on KEGG pathway annotation of the *C. reticulata* transcriptome, we totally identified 93 unigenes for the TAGBS pathway (Supplementary Table S11; Figure 4). Most of the genes involved in TAGBS pathway were present in the assembly, except for two *KCS* genes, *FATTY ACID ELONGATION 1* (*FAE1*, namely *KCS18*) and *KCS9* (Supplementary Table S11). We failed to identified *FAE1* and *KCS9* in the traditional camellia oil plant *C. oleifera*, based on previously published transcriptome dataset (Xia et al., 2014). *FAE1* is involved in the elongation of C_18_ to C_22_ FAs (Kunst et al., 1992), while *KCS9* is involved in the elongation of C_22_ to C_24_ FAs (Kim et al., 2013). *Arabidopsis fae1* mutant had a deficiency in producing very long-chain FAs (VLCFA) in seed oil (Kunst et al., 1992). Thus, the absence of *FAE1* might be an important factor contributing to the camellia oil with very low level of VLCFA.

**Figure 5:**
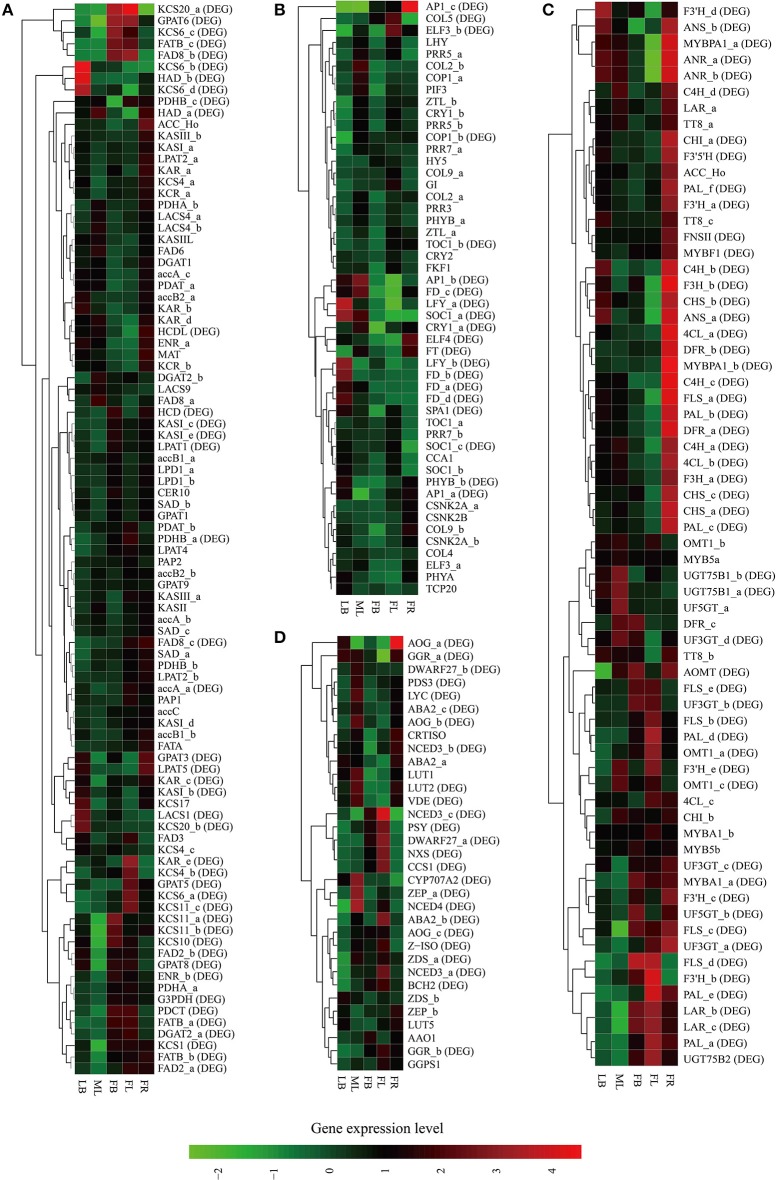
**Heat map representation and hierarchical clustering of putative genes involved in *C. reticulata*** flowering time control **(A)**, TAG biosynthesis **(B)**, flavonoid biosynthesis **(C)**, and carotenoid biosynthesis **(D)**. For gene abbreviations in Figures 4A–D, their full name and other information can be referenced in Supplementary Tables S11–S14, respectively. Expression profiles of these genes across five tissues (LB, leaf buds; ML, mature leaves; FB, flower buds; FL, flowers; FR, immature fruits; SE, seeds) are shown, and DE genes (fold change ≥ 4, FDR ≤ 0.001) are indicated as “DE” in brackets. Green and red colors are used to represent low-to-high expression levels, and color scales correspond to the mean centered log2-transformed FPKM values. A hierarchical cluster dendrogram is shown on the left.

The genes of this pathway showed high similarities to *Arabidopsis* with an average identity of 70% at amino acid sequence level, suggesting their potentially functional conservation. Many of these genes had multiple copies in *C. reticulata* compared with *Arabidopsis* (63 duplicated unigenes matched to 24 *Arabidopsis* unique homolog genes), such as *STEAROYL-ACP DESATURASE* (*SAD*, 3 unigenes), *FAD2* (2 unigenes), and *DGAT2* (2 unigenes). This phenomenon was also found in other *Camellia* species including *C. sinensis, C. taliensis*, and *C. oleifera* (data not shown). The results seemingly support the hypothesis that *Camellia* species might share whole genome duplication events during the course of evolution before their speciation (Shi et al., 2011). Gene expression profiles of the TAGBS pathway were shown in Figure 5A. For all of the 93 TAGBS unigenes, 42 were defined as DEGs (fold change = 4, FDR = 0.001). There are 29 (69%) DEGs up-regulated in flowers or flower buds, and some of them are FA desaturase homologs, including *SAD_a, FAD2_a, FAD2_b, FAD3, FAD8_b*, and *FAD8_c*. These results indicate that a variety of fatty acids could be demanded for flower development. FA desaturase genes are often related to the modification of membrane fluidity in response of cold hardiness (Kodama et al., 1994; Matteucci et al., 2011; Wang et al., 2013). The flowers of *C. reticulata* often bloom in winter when the temperatures are quite low (Supplementary Figures S6E,F, S7). Thus, high expression of these desaturase genes may explain the freezing tolerance of *C. reticulata* flowers and flower buds in winter.

Camellia oil contains 60–80% oleic acid and 5–10% linoleic acid. It is well characterized in *Arabidopsis* that plastid-localized enzymes were responsible for oleic acid biosynthesis with 18:0-ACP as precursor: SAD catalyzes the conversion of stearoyl-ACP to oleoyol-ACP (Shanklin and Somerville, 1991), while FATTY ACYL-ACP THIOESTERASE A (FATA) plays a key role in the formation of free oleic acid (Hellyer et al., 1992; Moreno-Perez et al., 2012). *FAD2* encodes an oleate desaturase that required for PUFA biosynthesis in endoplasmic reticulum (Okuley et al., 1994). In this study, *FATA* gene was observed with only one copy, while *SAD* and *FAD2* were observed with 3 and 2 copies, respectively (Supplementary Table S11). Different expression patterns were observed among the copies of *SAD* and *FAD2*. *FAD2_a* was up-regulated in young fruit, while *FAD2_b* was down-regulated in young fruit (Figure 5A). Clustering analysis of gene expression profiles shows that, *SAD_a* and *FATA* were co-expressed genes in the same subcluster, while *SAD_b* and *SAD_c* were in different subclusters (Figure 5A). Using qRT-PCR, we also investigated the expression levels of several TAGBS-related genes in ripening seeds. While *FAD2_a* had a lower expression level in seeds than in mature leaves (Figure 2E), *SAD_a* and *FATA* had several fold higher expression level in seeds than in mature leaves (Figures 2D,H). These observations suggest that *SAD_a* and *FATA* may be key genes responsible for MUFA production in *C. reticualta* seeds. Up-regulation of *SAD_a* gene and down-regulation of *FAD2_a* gene may be an efficient way to control the production ratio of MUFAs to PUFAs. Previously, Xia et al. (2014) found that parallel evolution of *FAD2* genes may occur between *Camellia* species and olive, which might result in the similar FA composition in their seed oil. Moreover, *FAD2* RNAi experiment in *Camelina sativa* showed evidence that *FAD2* is an efficient target for genetic manipulation toward lowering the PUFA content and increasing the oleic acid content (Nguyen et al., 2013). Our data here show that the transcription levels of *SAD, FAD2*, and *FATA* were highly regulated in seeds of *C. reticulata*. Their expression patterns may well explain why the FA composition of camellia oil was dominated by oleic acid but with relative low PUFA levels.

### Photoperiodic flowering pathway in *C. reticulata*

To better understand the flowering transition process in *C. reticulata*, we conducted a field study to record the timing series of major vegetative and reproductive events over an entire year (Supplementary Figures S6A–L). Our observation showed that the flowering transition of *C. reticulata* often occurs at the apex of branch around mid-April when the day length is more than 12 h in Sichuan, China (Supplementary Figures S6A, S7). This result suggests that the photoperiodic flowering pathway may determine flowering time control of *C. reticulata*.

In the model plant, *A. thaliana*, genes involved in photoperiodic flowering pathway were well characterized (Andres and Coupland, 2012). Homology searches successfully identified 50 unigenes potentially involved in this pathway (Supplementary Table S12). Of them, 36 (72%) candidate genes were represented with full-length CDSs, such as *CRYPTOCHROMEs* (*CRYs*), *LATE ELONGATED HYPOCOTYL* (*LHY*), *TIMING OF CAB EXPRESSION 1* (*TOC1*), *FKF1, GI, SOC1*, and *FT*. However, *CO* and its transcription repressor *CYCLING-DOF-FACTOR-1 (CDF1)* were absent in the assembly. *CO* was expressed at an extremely low level in *Arabidopsis* and was only actively detected in leaves or shoots under long-day condition (Putterill et al., 1995). Thus, the possibility cannot be excluded that the unique expression pattern of *CO* led to the lack of itself in our results. Alternatively, we found several homologs of the *CO* gene family (e.g., C*OL2, COL4, COL5*, and *COL9*; Supplementary Table S12). Similar to genes in TAGBS pathway, many genes involved in the photoperiodic flowering pathway appeared to have multiple copies in *C. reticulata*. For instances, *FD* was observed with 4 copies, and both *SOC1* and *AP1* were observed with three copies (Supplementary Table S12). Based on sequence comparisons, the candidate genes in this pathway showed moderate similarity to *Arabidopsis* with an average identity of 62% at amino acid sequence level. However, great sequence differences were found in *ELF3_a, ELF3_b*, and *LHY* with identities lower than 37%. The complex evolutionary process may bring about sequence variations and functional differentiation of these genes between *C. reticulata* and *Arabidopsis*.

The gene expression patterns of the photoperiodic flowering pathway genes across the tested tissues were shown in Figure 5B. A total of 20 putative genes were identified as DEGs, including *FT, SOC1* (*SOC1_*a and *SOC1_*c), *LFY* (*LFY_a* and *LFY_b*), and *AP1* (*AP1_a, AP1_b*, and *AP1_c*) (Figure 5B). The photoperiod and irradiance are mainly perceived by mature leaves. Nevertheless, many of these DEGs were not specially expressed in mature leaves. For examples, *SOC1_a* was up-regulated in mature leaves and leaf buds, and *FT* was up-regulated in mature leaves and young fruits (Figure 5B). These results suggest that these genes may play important roles in multiple developmental processes. The duplicated genes in this pathway demonstrate differential expression patterns across these tissues. For example, while *SOC1_a* was up-regulated in mature leaves and leaf buds, *SOC1_b* and *SOC1_c* were actively expressed in leaf buds and flowers (Figure 5B). Many studies suggested that the alteration of spatiotemporal expression is an important indicator of functional divergence in duplicated genes (Ganko et al., 2007). Thus, these duplicated genes may play diverse roles in multiple developmental processes.

### Flavonoid biosynthesis pathway in *C. reticulata*

**Figure 6:**
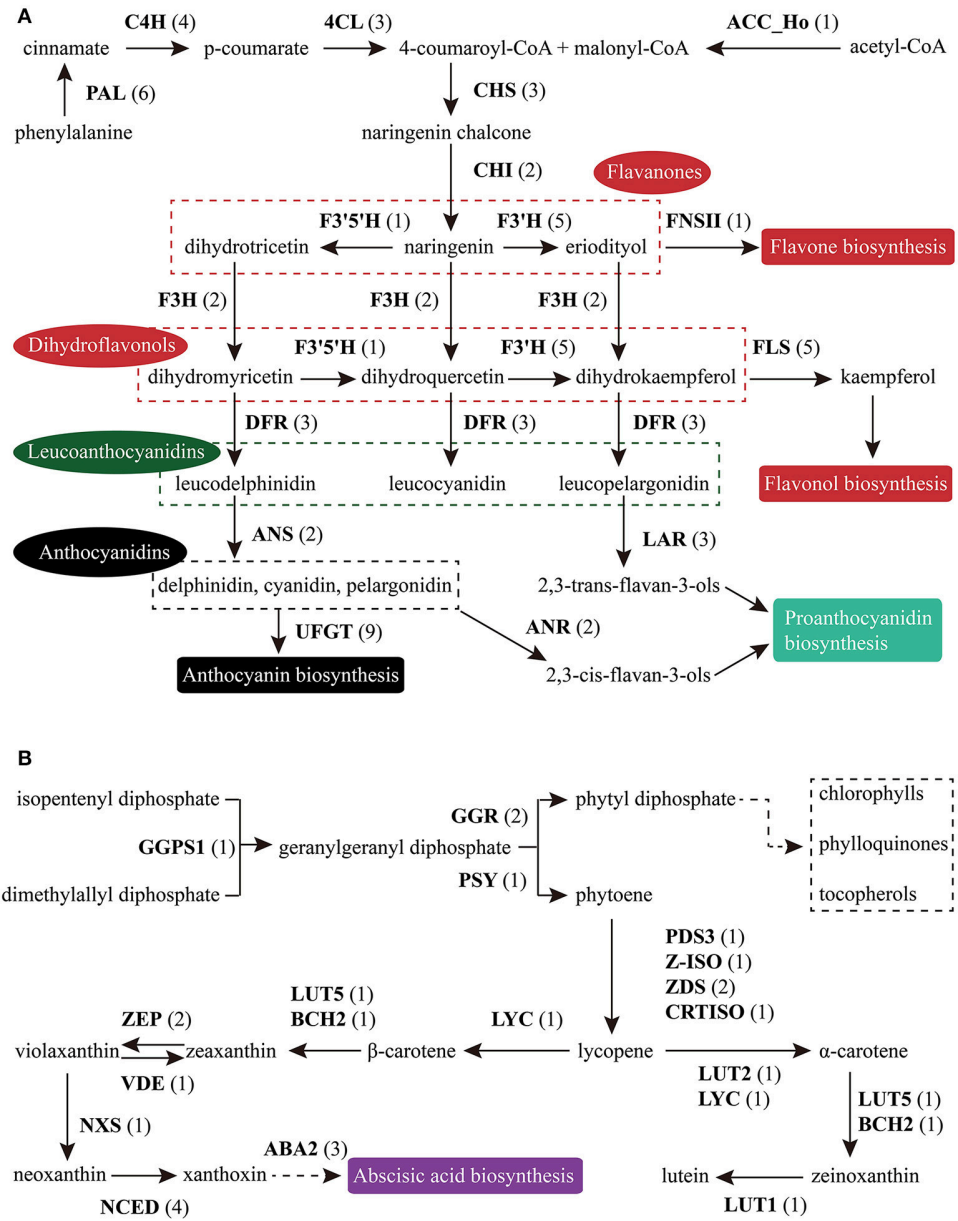
**Putative genes involved in FlaBS (A) and CrtBS (B)**. The numbers in brackets following each gene name indicate the number of unigenes annotated to that gene. For each gene abbreviation, its full name and other information can be checked in Supplementary Table S13, 14.

Flavonoids include the four major subgroups of flavones, flavonols, anthocyanins and PAs that play vital roles in flower coloration and many other plant physiological processes. Homology searches identified 56 unigenes as candidate genes that are related to the FlaBS pathway (Supplementary Table S13, Figure 6A). Most of these genes had multiple copies in *C. reticulata* compared to *Arabidopsis*, except for six genes (*ACC_Ho, FNSII, UGT75B2, AOMT, MYBF1*, and *F3*′*5*′*H*; Supplementary Table S13). Expression profiles for these genes were shown in Figure 5C. Overall, 89.3% (50/56) of these putative genes were defined as DEGs, indicating that most of these genes were extensively regulated at the level of transcription.

In grape, *VviMYB5* (*MYB5a* and *MYB5b*) (Deluc et al., 2006, 2008), *VviMYBF1* (Czemmel et al., 2009), *VviMYBPA1* (Bogs et al., 2007), and *VviMYBA1* (Kobayashi et al., 2004) were characterized as R2R3 MYB TF genes that play major roles in the production control of total flavonoids, flavonol, PAs, and anthocyanin pigments, respectively. The homologs of these TF genes were also identified in our analysis. Neither *MYB5a* nor *MYB5b* in *C. reticulata* is differentially expressed across tissues (Figure 5C), which is consistent to their putative roles as general regulators of flavonoid precursors (Deluc et al., 2006, 2008). *VviMYBF1* in grape has two functional MYB domains and serves as a specific regulator in expression control of *FLAVONOL SYNTHASE* (*FLS*) gene to regulate the flavonol production (Czemmel et al., 2009). The *C. reticulata MYBF1* was found with only a single MYB domain (Supplementary Figure S8), making great difference with its homolog *VviMYBF1*. Furthermore, none of five copies of *FLS* seems to be correlated with *MYBF1* at expression level, which is inconsistent with previous observation that *MYBF1* is a positive regulator of *FLS* gene (Czemmel et al., 2009). Thus, *MYBF1* in *C. reticulata* might have evolved a novel function, which was preferentially expressed in seeds (Figure 3J).

Two copies of *MYBPA1* were observed in the *C. reticulata* transcriptome, *MYBPA1_a* was a bit more conserved than *MYBPA1_b* compared to *VviMYBPA1* (Supplementary Table S13). While *MYBPA1_b* showed specific expression in young fruits, *MYBPA1_a* were highly expressed in leaf buds, mature leaves and young fruits. ANTHOCYANIDIN REDUCTASE (ANR) and LEUCOANTHOCYANIDIN REDUCTASE (LAR) are known as two key enzymes in the biosynthesis of PAs. While ANR catalyzes the formation of cis-flavan-3-ol (an oligomeric form of PAs) from anthocyanidin (Xie et al., 2003), LAR catalyzes the formation of tran-flavan-3-ol (another oligomeric form of PAs) from leucoanthocyanidin (Mauge et al., 2010). Previous report showed that *VviMYBPA1* positively activated the expression of *ANR* and *LAR* to specifically control PAs production (Bogs et al., 2007). In this study, the PA-specific biosynthetic gene *ANR* had two copies (*ANR_a* and *ANR_b*) in the assembly, and both of them had highly similar expression patterns with *MYBPA1_a* across tissues (Figures 3G,I, 4C). Among three copies of *LAR*, only *LAR_a* appeared high similar expression patterns with *MYBPA1_a*, which was also validated by qRT-PCR (Figures 3E,I). The expression patterns of *LAR_a, ANR_a*, and *ANR_b* correlated with that of *MYBPA1_a* suggest that the regulation of *C. reticulata MYBPA1_a* may be similar to its homologs in grape.

In this study, two copies of *MYBA1* were identified in the *C. reticulata* transcriptome. *MYBA1_a* was more conserved than *MYBA1_b* compared to *VviMYBA1* (Supplementary Table S13). *MYBA1_a* was identified as a DEG, which was highly expressed in flower buds, flowers and young fruits but lowly expressed in leaf buds and mature leaves (Figures 3K, 5C). Clustering analysis of gene expression profiles showed that *MYBA1_a* shared the same subcluster with several anthocyanin-specific biosynthetic genes, including *ANTHOCYANIDIN 3-O-GLUCOSYLTRANSFERASE* (*UF3GT_a* and *UF3GT_c*) and *ANTHOCYANIDIN 5-O-GLUCOSYLTRANSFERASE* (*UF5GT_b*). *MYBA1_a* and these anthocyanin-specific biosynthetic genes were highly expressed in both flower buds and flowers (Figures 3H,K, 5C), indicating their important roles of anthocyanin biosynthesis in flower developments.

### Carotenoid biosynthesis pathway in *C. reticulata*

Similar to flavonoids, carotenoids are a diverse group of colorful plant pigments. They play vital roles in many essential physiological processes such as the photosynthesis, flower coloration, and production of phytohormones (Cazzonelli and Pogson, 2010). In the assembly, we successfully identified 33 unigenes potentially involved in the CrtBS pathway (Figure 6B and Supplementary Table S14). Different from the FlaBS pathway, most (70%) of these genes in this pathway appeared a single copy. Seven genes had multiple copies, including *GERANYLGERANYL REDUCTASE* (*GGR*), *ZETA-CAROTENE DESATURASE* (*ZDS*), *ZEAXANTHIN EPOXIDASE* (*ZEP*), *NINE-CIS-EPOXYCAROTENOID DIOXYGENASE 3* (*NCED 3*), *ABA-GLUCOSYLTRANSFERASE* (*AOG*), *BETA-CAROTENE ISOMERASE* (*DWARF27*), and *XANTHOXIN DEHYDROGENASE* (*ABA2*) (Supplementary Table S14). The expression profiles of these CrtBS-related genes are shown in Figure 5D. Most (25/33) of these genes were defined as DEGs, and the majority of these DEGs were up-regulated in mature leaves or flowers (Figure 5D). Leaf tissues typically accumulate lutein, beta-carotene, violaxanthin, and neoxanthin for the photosynthesis, antenna assembly, and photoprotection (Cazzonelli and Pogson, 2010). Thus, genes related to the synthesis of these compounds are reasonably up-regulated in mature leafs, such as *GGR_a, LUTEIN1* (*LUT1*), *LUT2*, and *LYCOPENE CYCLASE* (*LYC*) (Figure 5D). *PHYTOENE SYNTHASE* (*PSY*), which encodes a rate-limiting enzyme that functions in the first committed step of the CrtBS pathway (Cazzonelli and Pogson, 2010), was markedly up-regulated in both flower buds and flowers (Figure 5D). Clustering analysis of gene expression profiles showed that *NEOXANTHIN SYNTHASE* (*NXS*) and *NCED3_c* had similar expression patterns with *PSY* (Figure 5D). In abscisic acid (ABA) biosynthesis, the NXS enzyme catalyzes the conversion of violaxanthin into neoxanthin (Al-Babili et al., 2000), and the NCED enzyme subsequently catalyzes the cleavage of neoxanthin, which represents the first committed step of ABA biosynthesis (Qin and Zeevaart, 1999). Previously, *ZEP* and *ABA2* in *Arabidopsis* were also identified as ABA biosynthetic genes (Gonzalez-Guzman et al., 2002). Figure 5D show that *ABA2_b, NCED4, NCED3a, ZEP_a* were actively expressed in the camellia flowers. Collectively, ABA biosynthesis genes may play important roles in *C. reticulata* flowers for developmental processes.

Carotenoids provide flowers with distinct colors, ranging from yellow to orange or red. In yellow-flowered *Camellia* species, such as *C. chrysantha*, carotenoids were reported to contribute to yellow color of its petal (Hwang et al., 1992; Nishimoto et al., 2004). Red-flowered *Camellia* species, such as *C. reticulata*, are reported to have high amounts of anthocyanins but few carotenoids (Li J. B. et al., 2008). As illustrated in Figure 5D, several genes directly related to the formation of pigment compounds (e.g., lutein, zeaxanthin, carotene), such as *LUT1, LUT2, LUT5*, and *LYC*, were expressed at relatively low levels in flower buds and flowers of *C. reticulata*. Low expression level of these genes in flower may account for a low level of carotenoid-based color compounds in camellia flower. These data also supports that it is the flavonoid biosynthesis pathway rather than carotenoids biosynthesis pathway playing dominant roles in *C. reticulata* flower coloration. Activation of these genes that involved in the synthesis of carotenoid-based color compounds might be useful in genetic manipulations of flower color in *Camellia* species in future.

## Conclusions

We first report the *de novo* assembly of the *C. reticulata* transcriptome. *De novo* assembly has provided a catalog of 141,460 unigenes with a total length of ~96.1 million nucleotides and an N50 of 1080 nt. Systematic evaluation indicated a good quality of the transcriptome assembly, which is suitable for further studies such as EST-SSRs mining and pathway analysis. We identified the majority of candidate genes related to TAGBS, FlaBS, CrtBS, and photoperiodic flowering pathways. The results also showed that FA desaturase genes were highly regulated, which may be related to cold hardiness response and FA composition control of camellia seed oil. Both the flaBS and CrtBS pathways were extensively regulated in *C. reticulata*. The former mainly participated in the biosynthesis and metabolism of proanthocyanidins, flavonols and anthocyanin pigments for flower color, while the latter play essential role in ABA synthesis rather than color maintenance. These useful resources will further enhance basic and applied researches on this economically important *Camellia* plant.

## Author contributions

LG designed the research. QY, HH, YT performed necessary experiments. QY, HH, YT, and EX performed bioinformatics analyses and interpreted the results. QY, HH, and LG wrote the paper. All authors read and approved the final manuscript.

### Conflict of interest statement

The authors declare that the research was conducted in the absence of any commercial or financial relationships that could be construed as a potential conflict of interest.

## Supplementary material

Supplementary Figure 1 **Tissues of wild diploid *C. reticulata* used for RNA-Seq (A–E) and qRT-PCR validation (A–F). (A)** leaf buds with length of 30–50 mm collected in March, 2012; **(B)** mature leaves collected in July, 2012; **(C)** flower buds with a diameter of 15–25 mm collected in December, 2012; **(D)** fully opened flowers collected in December, 2012; **(E)** immature fruits with a diameter of 20–30 mm collected in March, 2012; **(F)** blackening seeds collected in July, 2013.

Supplementary Figure 2 **Identity and coverage distribution of the best BLAST hits for each unigene against the *Camellia* EST database**. We used megaBLAST (*E* ≤ 10^−9^) to search against the local *Camellia* EST database and extracted the best hit of each sequence for analysis. The green line indicates identity distribution of the best match, while the red line shows the coverage distribution of the best match.

Supplementary Figure 3 **GO Classification of the *C. reticulata* transcriptome**. GO terms assigned to each unigenes by BLAST2GO are summarized into three main GO categories (biological process, cellular component, molecular function) and 47 subcategories using the web-based tool WEGO.

Supplementary Figure 4 **Polymorphism survey of SSR markers with 24 individual *C. reticulata* plants. (A)** Genomic DNA extracted from 24 individuals using the modified CTAB method. Genomic DNA resolved by electrophoresis on 1% agarose gel and visualized with ethidium bromide staining. Lanes 1–24: 24 individuals; M: 1-kb DNA ladders. **(B–H)** Polymorphism survey results showed that 7 SSR loci were polymorphic. PCR products were resolved by electrophoresis on 10% non-denaturing polyacrylamide gels and visualized by silver staining. Lanes 1–24: 24 individuals; M: 50-bp DNA ladders.

Supplementary Figure 5 **Expression characteristics of the expressed unigenes. (A)** Venn diagram shows the presence of the expressed unigenes among five tissue types. **(B)** Cumulative distributions of average expression levels of the expressed unigenes in the five tested tissues. The average cumulative abundance of unigenes across the five tested tissues was calculated by sorting unigenes according to their descending expression levels. The cumulative values of expression levels and unigene number are displayed as a percentage of overall expression level values and total unigenes, respectively.

Supplementary Figure 6 **Major seasonal development (vegetative and reproductive) events of *C. reticulata*. (A)** Flowering began after the formation of a new branch (mid-April); **(B)** the floral primordial began to form a new flower bud (late-April); **(C,D)** flower bud growth in June and October respectively; **(E)** the first camellia flower began to bloom in late November; **(F)** a flower would last ~5 days and then faded away; **(G)** fruiting began when the flower faded away; **(H)** growth of young fruit and leaf bud (mid-March); **(I)** leaf bud breaking the sheaths (late-March); **(J)** continued young fruit growth while the leaf bud began to form a new branch (late-March to mid-April); **(K)** continued young fruit growth (June); **(L)** a fully ripened fruit splitting the capsule from the top (mid-August to late-September).

Supplementary Figure 7 **The relationship between the weather and *C. reticulata* reproductive phenology**. The data shown here are based on the daily report from Weather China (http://www.weather.com.cn/).

Supplementary Figure 8 **Comparison of the conserved domains between *C. reticulata MYBF1* and *VviMYBF1.*** Conserved domains were detected by BLASTp searches against the NR database on the NCBI web site. Domain hits with an E-value threshold of 0.001 are listed.

Supplementary Table 1 **Details of 24 *C. reticulata* individuals used in this study**.

Supplementary Table 2 **The putative genes and corresponding primers used for qRT-PCR analysis**.

Supplementary Table 3 **Details of 20 EST-SSRs used in the polymorphism survey of *C. reticulata***.

Supplementary Table 4 **The top 10 assembled unigenes examined for chimerical assembly errors**.

Supplementary Table 5 **Sequence homology of the *C. reticulata* unigenes**. Top BLAST hits (*E* ≤ 10^−3^) from five databases (Swiss-Prot, NR, TrEMBL, TAIR10, and grape protein database) for all the unigenes are shown.

Supplementary Table 6 **The 10 most abundant PFAM families/domains for *C. reticulata* unigenes**.

Supplementary Table 7 **GO annotation, KO assignment and KEGG pathway annotation for *C. reticulata* unigenes**. GO terms were obtained based on the NR database annotations using Blast2GO software.

Supplementary Table 8 **List of SSR motifs and their frequencies in the unigenes of *C. reticulata***.

Supplementary Table 9 **List of DE unigenes and their expression values**. DE unigenes were assigned to 5 groups (DELB, DEML, DEFB, DEFL, and DEFR) based on their tissues where their highest expression occurred.

Supplementary Table 10 **KEGG pathway and GO enrichment analysis of DE unigenes**.

Supplementary Table 11 **Detailed information of putative genes related to TAG biosynthesis in the *C. reticulata* transcriptome**.

Supplementary Table 12 **Detailed information of putative genes related to photoperiodic flowering pathway in the *C. reticulata* transcriptome**.

Supplementary Table 13 **Detailed information of putative genes related to flavonoid biosynthesis in the *C. reticulata* transcriptome**.

Supplementary Table 14 **Detailed information of putative genes related to carotenoid biosynthesis in the *C. reticulata* transcriptome**.

## Abbreviations

ABA: abscisic acid
CDS: coding DNA sequence
CrtBS: carotenoid biosynthesis
CTAB: cetyl trimethylammonium bromide
DE: differentially expressed
EST: expressed sequence tag
FA: fatty acid
FDR: false discovery rate
flaBS: flavonoid biosynthesis
FPKM: expected number of fragments per kilobase of transcript sequence per millions base pairs sequenced
GO: gene ontology
KEGG: Kyoto Encyclopedia of Genes and Genomes
Knt: kilo-nucleotides
KO: KEGG orthology
Mnt: million nucleotides
MUFA: monounsaturated fatty acid
NCBI: National Center for Biotechnology Information
NR: the non-redundant protein database
PA: proanthocyanidin
PUFA: polyunsaturated fatty acid
qRT-PCR: quantitative reverse transcription polymerase chain reaction
RNA-Seq: RNA sequencing
SFA: saturated fatty acid
SSR: simple sequence repeat
Swiss-Prot: UniProtKB/Swiss-Prot
TAGBS: triacylglycerol biosynthesis
TAIR10: *A*.thaliana proteins database
TrEMBL: UniProtKB/TrEMBL
UFA: unsaturated fatty acid.
