# Supplementary figures and images for: Transcriptome Analysis Identifies Candidate Genes Related to Triacylglycerol and Pigment Biosynthesis and Photoperiodic Flowering in the Ornamental and Oil-Producing Plant, Camellia reticulata (Theaceae)
Source: Front Plant Sci. 2016 Feb 23;7:163. doi: 10.3389/fpls.2016.00163 (PMC4763035; doi:10.3389/fpls.2016.00163)

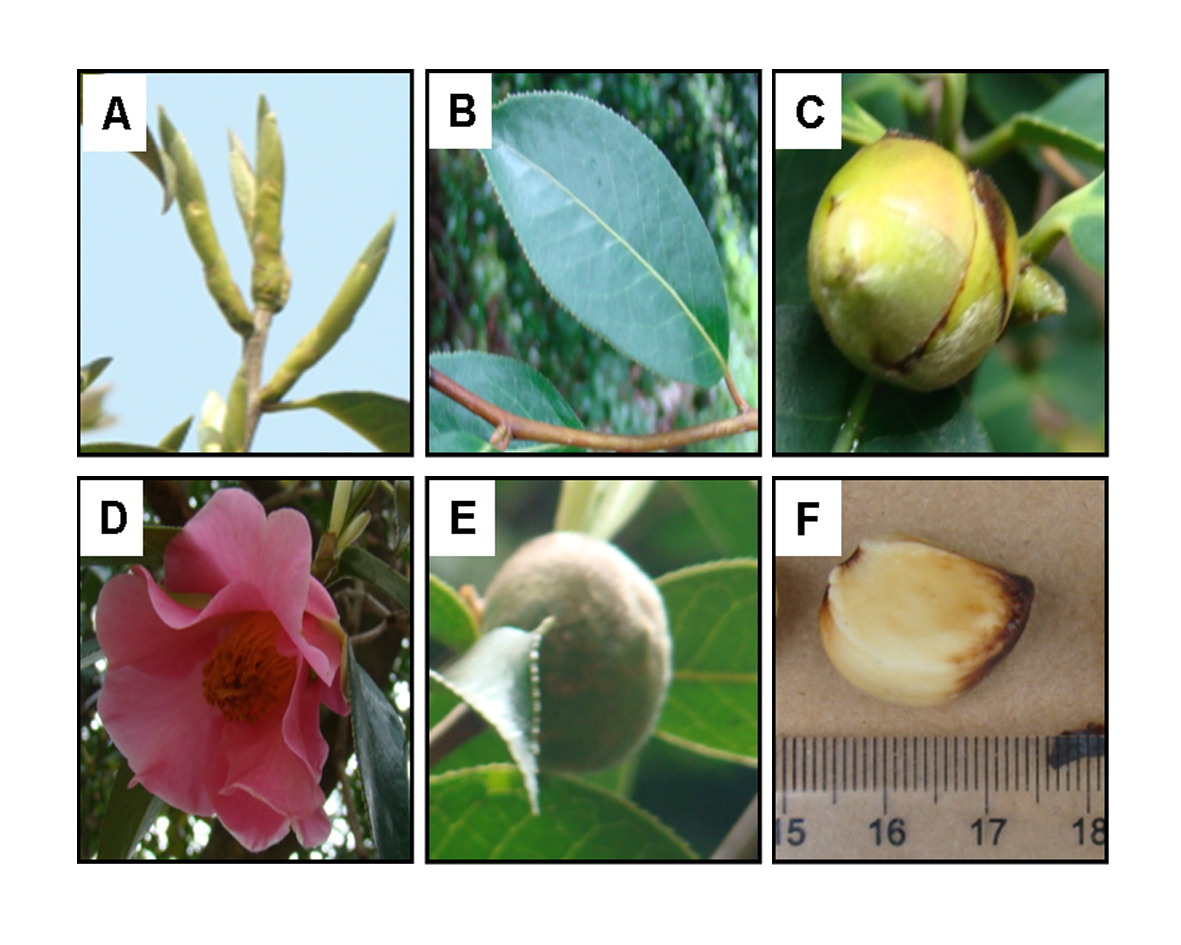

Supplement: Supplementary Figure 1 — Tissues of wild diploid C. reticulata used for RNA-Seq (A–E) and qRT-PCR validation (A–F). (A) leaf buds with length of 30–50 mm collected in March, 2012; (B) mature leaves collected in July, 2012; (C) flower buds with a diameter of 15–25 mm collected in December, 2012; (D) fully opened flowers collected in December, 2012; (E) immature fruits with a diameter of 20–30 mm collected in March, 2012; (F) blackening seeds collected in July, 2013. [file Image1.TIF]

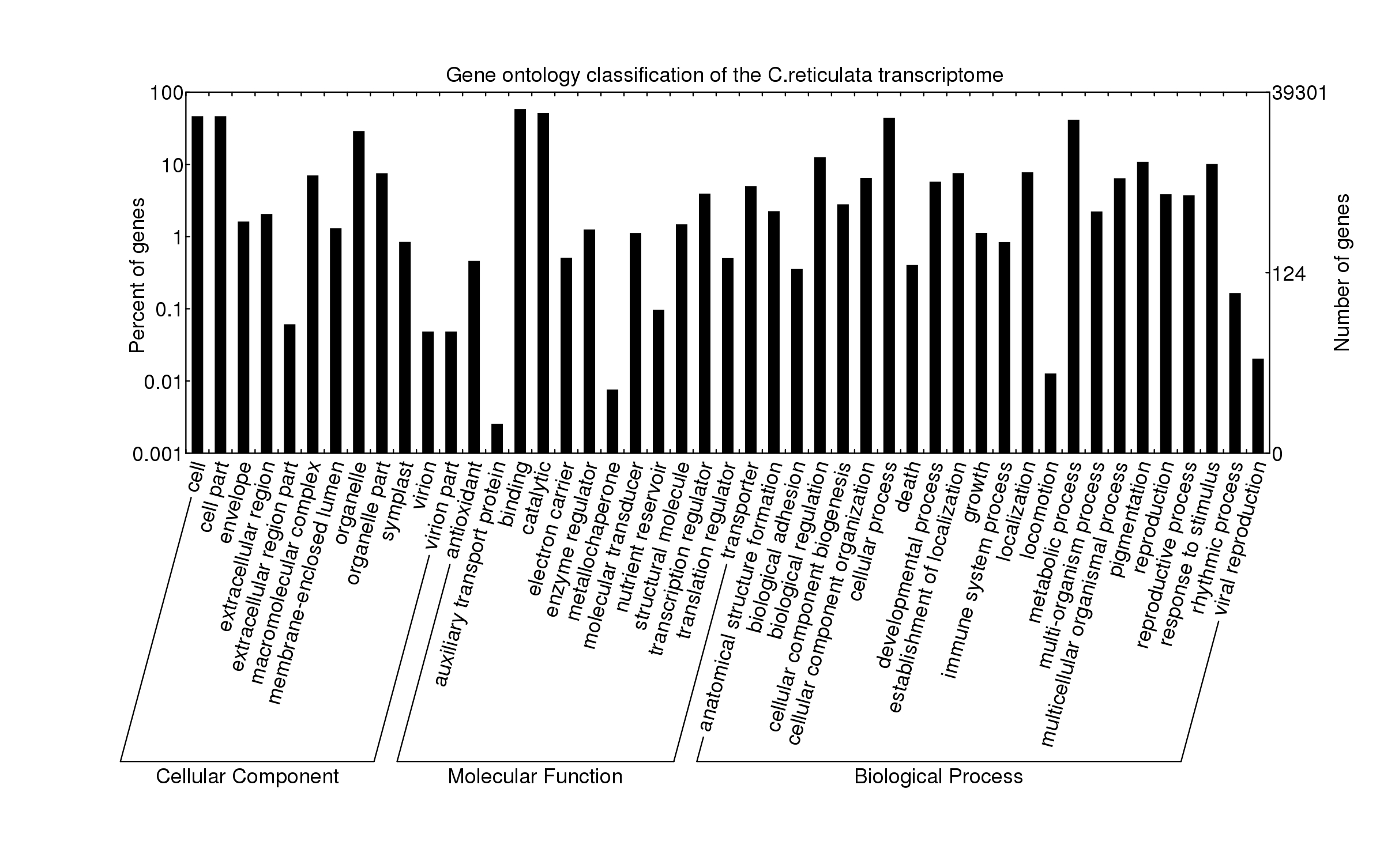

Supplement: Supplementary Figure 3 — GO Classification of the C. reticulata transcriptome. GO terms assigned to each unigenes by BLAST2GO are summarized into three main GO categories (biological process, cellular component, molecular function) and 47 subcategories using the web-based tool WEGO. [file Image3.JPEG]

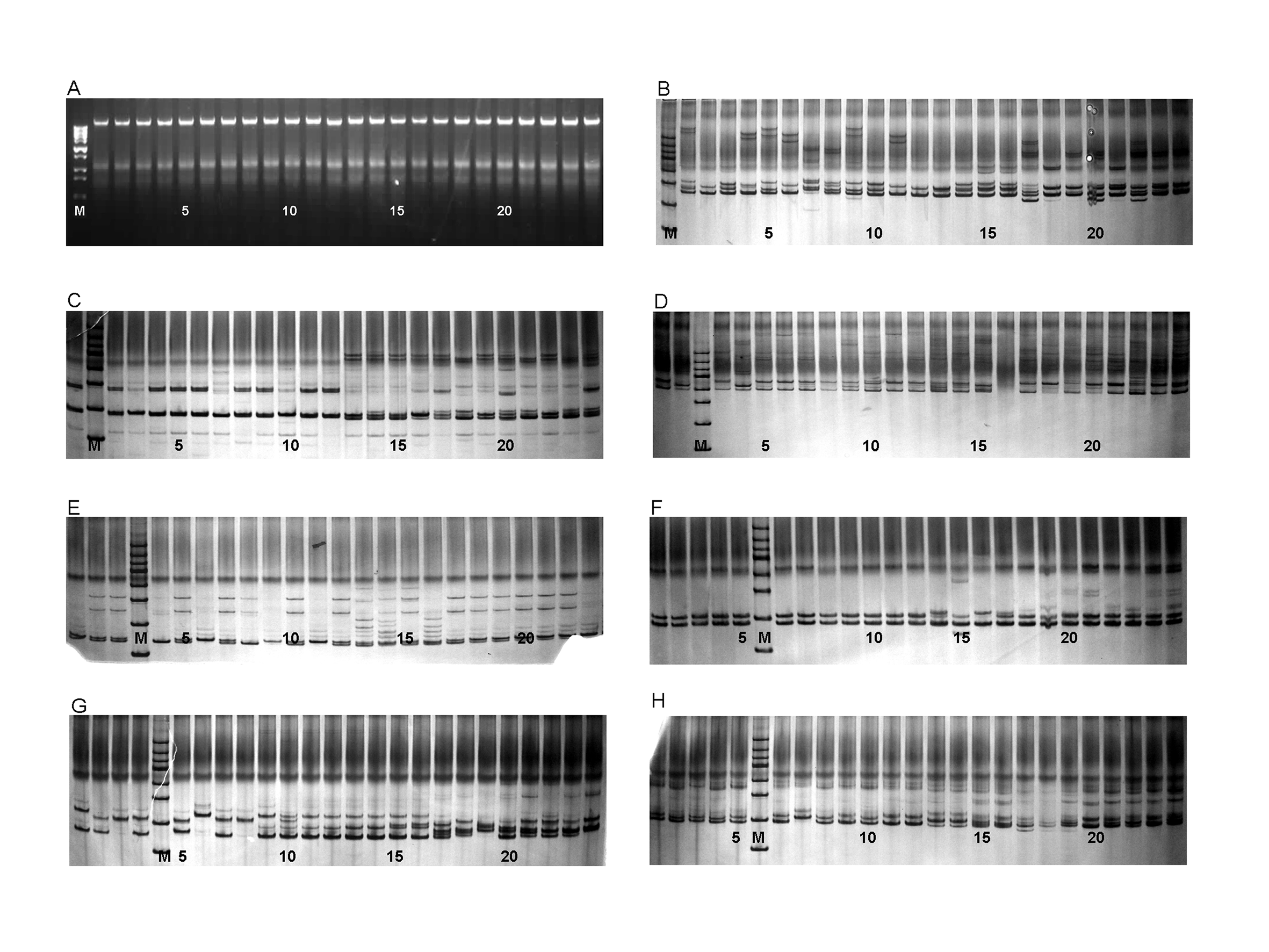

Supplement: Supplementary Figure 4 — Polymorphism survey of SSR markers with 24 individual C. reticulata plants. (A) Genomic DNA extracted from 24 individuals using the modified CTAB method. Genomic DNA resolved by electrophoresis on 1% agarose gel and visualized with ethidium bromide staining. Lanes 1–24: 24 individuals; M: 1-kb DNA ladders. (B–H) Polymorphism survey results showed that 7 SSR loci were polymorphic. PCR products were resolved by electrophoresis on 10% non-denaturing polyacrylamide gels and visualized by silver staining. Lanes 1–24: 24 individuals; M: 50-bp DNA ladders. [file Image4.TIF]

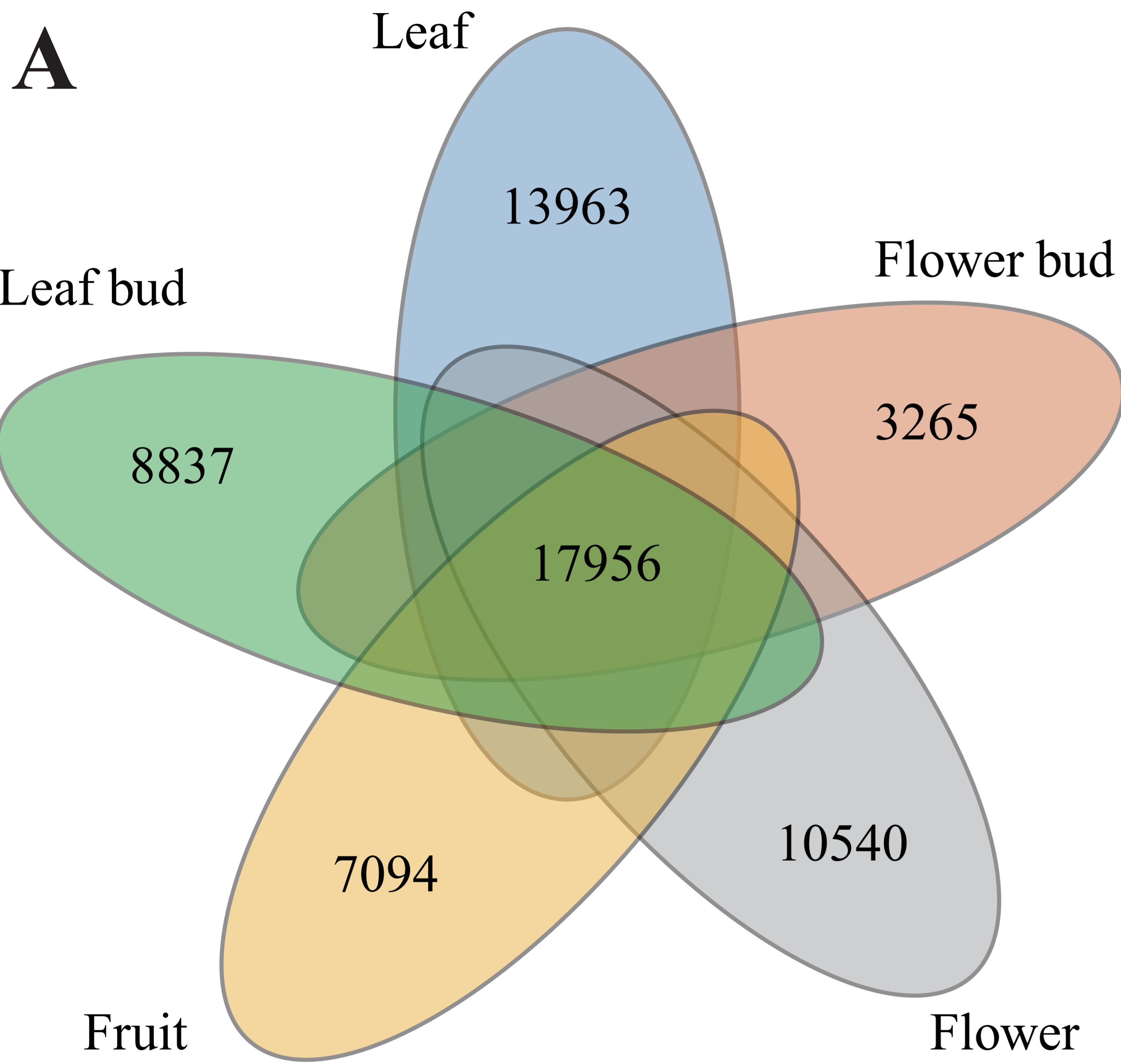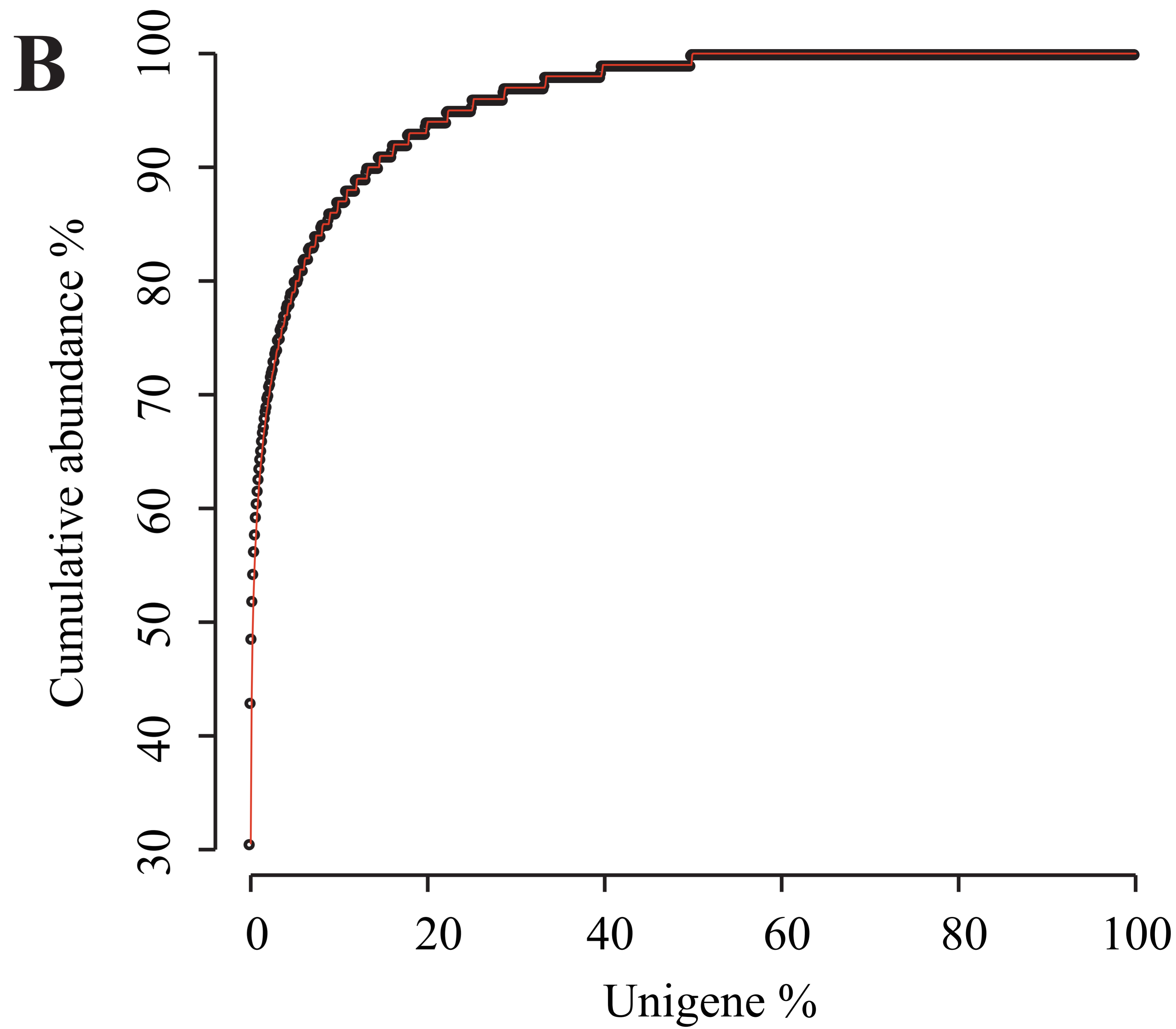

Supplement: Supplementary Figure 5 — Expression characteristics of the expressed unigenes. (A) Venn diagram shows the presence of the expressed unigenes among five tissue types. (B) Cumulative distributions of average expression levels of the expressed unigenes in the five tested tissues. The average cumulative abundance of unigenes across the five tested tissues was calculated by sorting unigenes according to their descending expression levels. The cumulative values of expression levels and unigene number are displayed as a percentage of overall expression level values and total unigenes, respectively. [file Image5.PDF]

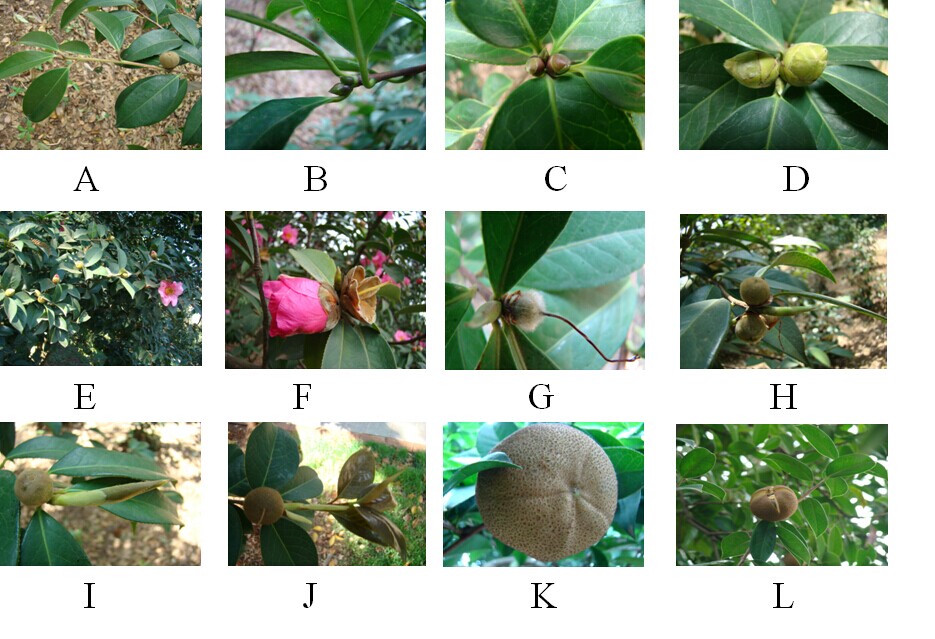

Supplement: Supplementary Figure 6 — Major seasonal development (vegetative and reproductive) events of C. reticulata. (A) Flowering began after the formation of a new branch (mid-April); (B) the floral primordial began to form a new flower bud (late-April); (C,D) flower bud growth in June and October respectively; (E) the first camellia flower began to bloom in late November; (F) a flower would last ~5 days and then faded away; (G) fruiting began when the flower faded away; (H) growth of young fruit and leaf bud (mid-March); (I) leaf bud breaking the sheaths (late-March); (J) continued young fruit growth while the leaf bud began to form a new branch (late-March to mid-April); (K) continued young fruit growth (June); (L) a fully ripened fruit splitting the capsule from the top (mid-August to late-September). [file Image6.JPEG]

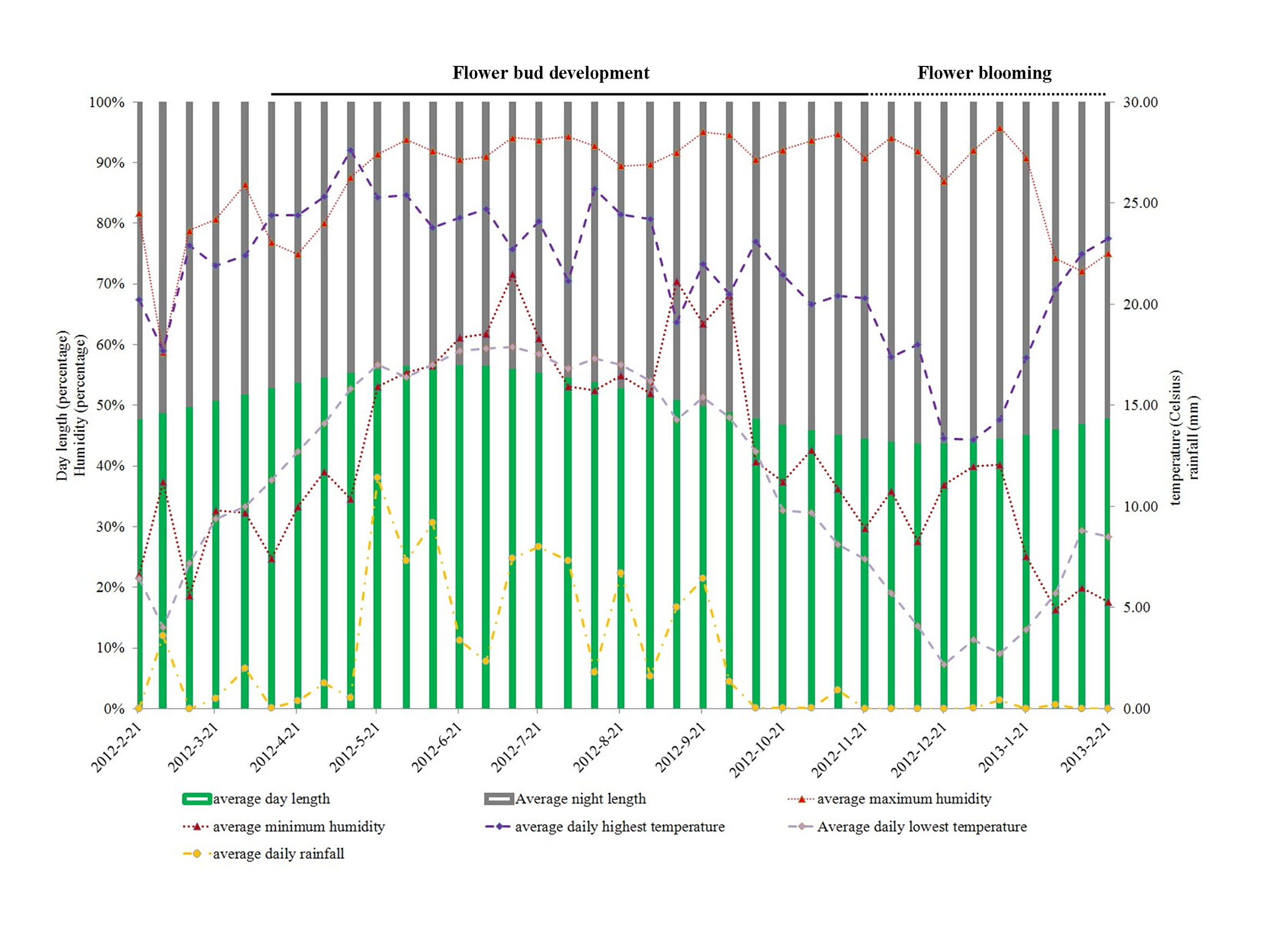

Supplement: Supplementary Figure 7 — The relationship between the weather and C. reticulata reproductive phenology. The data shown here are based on the daily report from Weather China (http://www.weather.com.cn/). [file Image7.TIF]

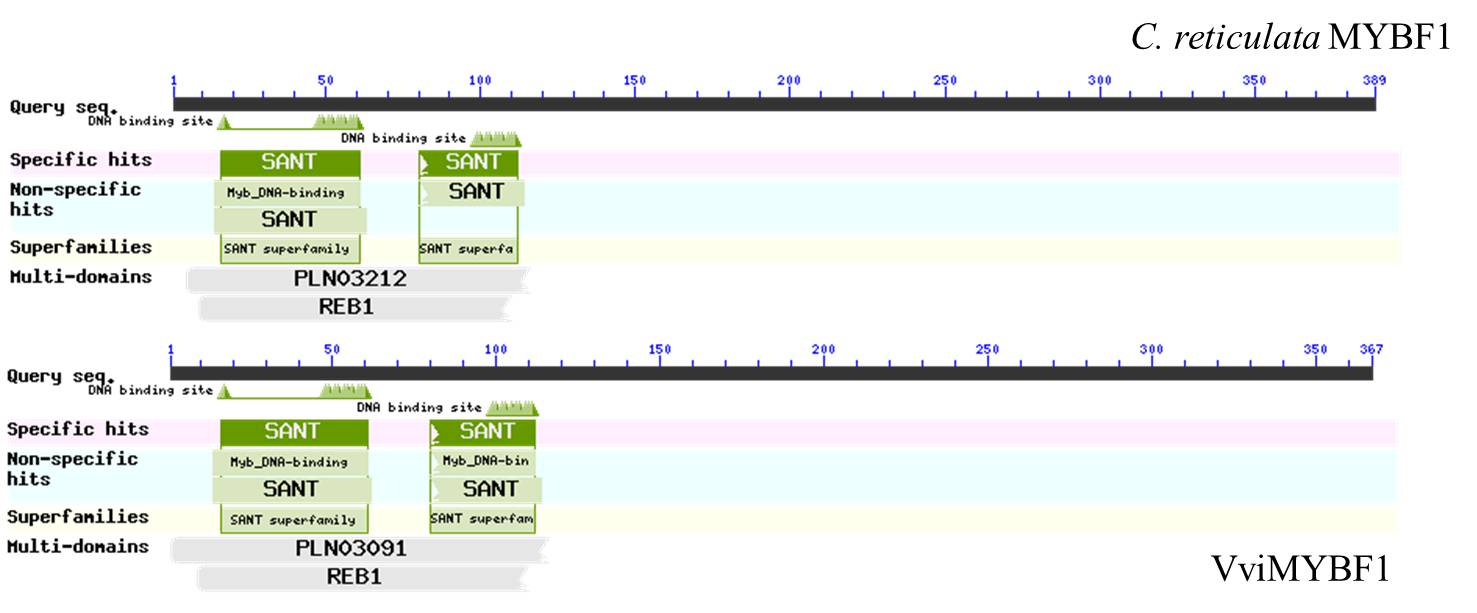

Supplement: Supplementary Figure 8 — Comparison of the conserved domains between C. reticulata MYBF1 and VviMYBF1. Conserved domains were detected by BLASTp searches against the NR database on the NCBI web site. Domain hits with an E-value threshold of 0.001 are listed. [file Image8.JPEG]
