# Supplementary material for: Transcriptome Analysis Identifies Candidate Genes Related to Triacylglycerol and Pigment Biosynthesis and Photoperiodic Flowering in the Ornamental and Oil-Producing Plant, Camellia reticulata (Theaceae)
Source: Front Plant Sci. 2016 Feb 23;7:163. doi: 10.3389/fpls.2016.00163 (PMC4763035; doi:10.3389/fpls.2016.00163)
Supplement: Supplementary Table 1 — Details of 24 C. reticulata individuals used in this study. [file Table1.DOC]

**Supplementary Table S1 Details of 24 *C. reticulata* individuals used in this study.**

| **No.** | **Name** | **Variety Type** | **Flower types*** | **Origin** |
| --- | --- | --- | --- | --- |
| 1 | YB1-1** | Wild | Single | Yanbian County, Sichuan Province |
| 2 | YB1-2 | Wild | Single | Yanbian County, Sichuan Province |
| 3 | YB1-3 | Wild | Single | Yanbian County, Sichuan Province |
| 4 | YB1-4 | Wild | Single | Yanbian County, Sichuan Province |
| 5 | YB1-5 | Wild | Single | Yanbian County, Sichuan Province |
| 6 | YB1-6 | Wild | Single | Yanbian County, Sichuan Province |
| 7 | YB1-7 | Wild | Single | Yanbian County, Sichuan Province |
| 8 | YB1-8 | Wild | Single | Yanbian County, Sichuan Province |
| 9 | YB1-9 | Wild | Single | Yanbian County, Sichuan Province |
| 10 | YB1-10 | Wild | Single | Yanbian County, Sichuan Province |
| 11 | YB1-11 | Wild | Single | Yanbian County, Sichuan Province |
| 12 | YB1-12 | Wild | Single | Yanbian County, Sichuan Province |
| 13 | YB2-1** | Wild | Single | Yanbian County, Sichuan Province |
| 14 | YB2-2 | Wild | Single | Yanbian County, Sichuan Province |
| 15 | YB2-3 | Wild | Single | Yanbian County, Sichuan Province |
| 16 | YB2-4 | Wild | Single | Yanbian County, Sichuan Province |
| 17 | Honghuayoucha 1 | Garden variety | Single | Kunming Botanical Garden, Kunming , Yunnan Province |
| 18 | Dali Cha | Garden variety | Fully Double,  Peony type | Kunming Botanical Garden, Kunming , Yunnan Province |
| 19 | Juban | Garden variety | Fully Double,  Rose type | Kunming Botanical Garden, Kunming , Yunnan Province |
| 20 | Honghuayoucha 2 | Garden variety | Single | Kunming Botanical Garden, Kunming , Yunnan Province |
| 21 | Zipao | Garden variety | Fully Double,  Rose type | Kunming Botanical Garden, Kunming , Yunnan Province |
| 22 | Zaotaohong | Garden variety | Semi-Double, Butterfly wing | Kunming Botanical Garden, Kunming , Yunnan Province |
| 23 | Shizitou | Garden variety | Fully Double,  Peony type | Kunming Botanical Garden, Kunming , Yunnan Province |
| 24 | Songzilin | Garden variety | Fully Double,  Rose type | Kunming Botanical Garden, Kunming , Yunnan Province |

* Flower types could be divided into 3 group (single flower, semi-double, fully double) and 8 types (trumpet, magnolia, lotus, wavy, butterfly wing, rose, radiate and peony), which have been described previously (Yu and Bartholomew, 1980).

** Voucher specimens (deposit number “Xiao 0001” for YB1 individuals and “Xiao 0010” for YB2 individuals) of wild type *C. reticulata* are deposited in the Herbarium of the Kunming Institute of Botany .

NOTE: YB1-2 was used for RNA sequencing and qRT-PCR validation. All of the 24 *C. reticulata* individuals were used for polymorphism survey.

**Reference:**

Yu T.T., Bartholomew B., The origin and classification of the garden varieties of Camellia reticulata, American Camellia Yearbook, 1980: 112-142.

Liu, L.Q. and Gu, Z.J., 2011. Genomic in situ hybridization identifies genome donors of Camellia reticulata (Theaceae). Plant Sci 180, 554-559.
