# Supplementary material for: Transcriptome Analysis Identifies Candidate Genes Related to Triacylglycerol and Pigment Biosynthesis and Photoperiodic Flowering in the Ornamental and Oil-Producing Plant, Camellia reticulata (Theaceae)
Source: Front Plant Sci. 2016 Feb 23;7:163. doi: 10.3389/fpls.2016.00163 (PMC4763035; doi:10.3389/fpls.2016.00163)
Supplement: Supplementary Table 2 — The putative genes and corresponding primers used for qRT-PCR analysis. [file Table2.DOC]

**Supplementary Table S2 The putative genes and corresponding primers used for qRT-PCR analysis.**

| **Gene name** | **Abbreviation** | **Assembly ID** | **Forward/Reverse primer** | **Product size (bp)** | **Homologs*** |
| --- | --- | --- | --- | --- | --- |
| Elongation factor 1 alpha | *EF1A* | CRD_FL_c48815_g1_i3 | 5’GATGCTACCTGACCATGCAGAAG3’  5’TCCCGACAGTGTTGATTTTCC3’ | 91 | AT5G10630, *Arabidopsis thaliana* |
| Microsomal omega-6 fatty acid desaturase | *FAD2_a* | CRD_FL_c46909_g3_i1 | 5’ACCATGCAATGGAAGCCACAAAAG3’  5’ACACCTTTGGTTTGGTCACTTTCG3’ | 148 | AT3G12120, *Arabidopsis thaliana* |
| Plastidial omega-6 fatty acid desaturase | *FAD6* | CRD_FR_c33808_g1_i1 | 5’GCCTTGGATGTCTATTGCTCACT3’  5’GCAATGACACAAGCCAAGCTTA3’ | 105 | AT4G30950, *Arabidopsis thaliana* |
| Acyl acyl-carrier-protein desaturase | *SAD_a* | CRD_FB_c39507_g9_i1 | 5’AGGGCAAAGGAGATTCCAGATG3’  5’CCTGTTTCATCCCGAACACCA3’ | 122 | AT2G43710, *Arabidopsis thaliana* |
| Fatty acyl-ACP thioesterase A | *FATA* | CRD_FL_c49868_g1_i1 | 5’TGCAACAACCCTTACCATGAGA3’  5’CACATCACTCCAAGCTGGGTATT3’ | 94 | AT3G25110, *Arabidopsis thaliana* |
| Homomeric acetyl-CoA carboxylase | *ACC_Ho* | CRD_FR_c31830_g4_i2 | 5’CCAGAGAGCGGTTTTCAGTATGTC3’  5’CCATCGGGTTTCTCCACTTGA3’ | 108 | AT1G36160, *Arabidopsis thaliana* |
| Heteromeric acetyl-CoA carboxylase biotin carboxylase subunit | *accC* | CRD_FR_c31599_g3_i1 | 5’TTGGAGAGCGTGATTGCAGTAT3’  5’CTGCATCACCCATGGCTTTC3’ | 105 | AT5G35360, *Arabidopsis thaliana* |
| beta-ketoacyl-ACP synthase II | *KAS II* | CRD_FB_c38830_g3_i3 | 5’CGCAGACAGAGACGCTTCAAT3’  5’GTCACAACTACTCGCCTTTGCTT3’ | 125 | AT1G74960, *Arabidopsis thaliana* |
| Plastidial omega-3 fatty acid desaturase | *FAD8_a* | CRD_FR_c33446_g2_i1 | 5’GGTTTTGTAATGTGGCTGGATCTC3’  5’TCCATTCCTTTCCACGATACCA3’ | 88 | AT5G05580, *Arabidopsis thaliana* |
| Acyl-CoA : diacylglycerol acyltransferase 1 | *DGAT1* | CRD_FR_c26741_g1_i1 | 5’CATTGCTGTTCCCTGCCACATATTC3’  5’GGTTGACCCAGAATGCTGAAAAAGC3’ | 159 | AT2G19450, *Arabidopsis thaliana* |
| Acyl-CoA : diacylglycerol acyltransferase 2 | *DGAT2_a* | CRD_FL_c32406_g1_i1 | 5’TACCCTATCACCGTCCAATGC3’  5’TGACCGTGTACTTCACTCACCTCTT3’ | 100 | AT3G51520, *Arabidopsis thaliana* |
| Phospholipid: diacylglycerol acyltransferase | *PDAT_a* | CRD_FR_c31766_g1_i2 | 5’GTTTGGGCAGTTCTGATTGCTA3’  5’CCGCACCTCAGTGTTCTGAAAT3’ | 108 | AT5G13640, *Arabidopsis thaliana* |
| R2R3-MYB transcription factor MYBPA1 | *MYBPA1_a* | CRD_FR_c34441_g7_i1 | 5’ATGTGGAAAGAGTTGCAGGCTAAG3’  5’CGGTTGCCAAGAAGAGCATG3’ | 120 | CAJ90831, *Vitis vinifera* |
| R2R3-MYB transcription factor MYBF1 | *MYBF1* | CRD_FR_c33684_g6_i5 | 5’TCTGACTTGAAGAGAGGCAACATATC3’  5’GTTGTCTGTTCTTCCTGGCAAGTG3’ | 120 | ACV81697, *Vitis vinifera* |
| R2R3-MYB transcription factor MYBA1 | *MYBA1_a* | CRD_FB_c32364_g1_i3 | 5’GAGAAGGAAAGTGGCACCAAGT3’  5’CCTCTCTTAATATTGGGCCTCAGA3’ | 106 | BAD18977, *Vitis vinifera* |
| Chalcone synthase | *CHS_a* | CRD_FR_c33206_g1_i3 | 5’GGAAGCATTCCAACCCTTGG3’  5’GTGGCCCGTAGCTTCTCTTC3’ | 129 | AT5G13930, *Arabidopsis thaliana* |
| Flavonol synthase | *FLS_a* | CRD_FR_c39460_g1_i1 | GTACCCACCATGCCCACAAC3’  CCCAATTACCGTCTTTCCAAAC3’ | 125 | AT5G08640, *Arabidopsis thaliana* |
| Flavonol synthase | *FLS_b* | CRD_FB_c35880_g3_i1 | ATCGTACAGGGAAGCCAATGAA3’  CTAGTGACAGCCACCGGAACA3’ | 80 | AT5G08640, *Arabidopsis thaliana* |
| Leucoanthocyanidin reductase | *LAR_a* | CRD_FR_c27159_g2_i1 | TATGGTGATGGCAGTGTCAAAGC3’  GCAGGATGGTCGGAAATGAAC3’ | 120 | ADZ58167, *Camellia sinensis* |
| Leucoanthocyanidin reductase | *LAR_b* | CRD_FL_c51519_g9_i3 | GGGCATGACATAGATAGAGCTGATC3’  TCCTCACCATTCGCTTTTCTTTG3’ | 73 | CAI56323, *Gossypium arboreum* |
| Anthocyanidin reductase | *ANR_a* | CRD_FR_c31473_g1_i1 | CCTGGTTTTCCATGTCGCTACA3’  GTTCCTGCTTTTGCACAAGCTT3’ | 120 | AT1G61720, *Arabidopsis thaliana* |
| Anthocyanidin 3-O-glucosyltransferase | *UF3GT_a* | CRD_FR_c2277_g1_i1 | CGGTGGCATTTGGGAAAGTG3’  CACCCACAATGTGTCACAAACAC3’ | 91 | AT5G17050, *Arabidopsis thaliana* |
| Anthocyanidin synthase | *ANS_a* | CRD_FL_c52432_g4_i2 | TCCCTGAAGACAAGCGTGACA3’  AGGGCCGACAGGACTTTTGTT3’ | 118 | AT4G22880, *Arabidopsis thaliana* |

* Gene homolog in *Arabidopsis* protein database can be retrieved with its gene locus ID, while gene homolog in NR database can be retrieved with its accession ID.
