# Supplementary material for: Transcriptome Analysis Identifies Candidate Genes Related to Triacylglycerol and Pigment Biosynthesis and Photoperiodic Flowering in the Ornamental and Oil-Producing Plant, Camellia reticulata (Theaceae)
Source: Front Plant Sci. 2016 Feb 23;7:163. doi: 10.3389/fpls.2016.00163 (PMC4763035; doi:10.3389/fpls.2016.00163)
Supplement: Supplementary Table 3 — Details of 20 EST-SSRs used in the polymorphism survey of C. reticulata. [file Table3.DOC]

**Supplementary Table S3 Details of 20 EST-SSRs used for polymorphism survey of *C. reticulata*.**

| **Primer** | **Locus** | **Annotation** | **Location** | **Primer sequence (5’-3’)** | **Repeat motif** | **Tm (℃)** | | **Expected size (bp)** | **PCR products** |
| --- | --- | --- | --- | --- | --- | --- | --- | --- | --- |
| SSR1 | CRD_FL_c41530_g1_i1 | polyamine oxidase 2 | 5’ UTR | F: TGGCCGATCGGGATTCAAT  R: TGACCAAACAGCTCATGCG | (GT)4GC(GT)6 | 58 | | 176 | E |
| SSR 2 | CRD_ML_c88806_g5_i13 | DEAD/DEAH box RNA helicase family protein | 5’ UTR | F: CCACAAGTAGGAAAGCCCCT  R: TACGGACGGCATTCTGTGAG | (CTT)5 | 57 | | 242 | E |
| SSR 3 | CRD_FR_c27989_g1_i1 | chalcone-flavanone isomerase | CDS | F: GTGTTTCCCCAGCAGCAAAG  R: TCAGCAACTTAAGCACTCAC | (GCT)6 | 53 | | 142 | E |
| SSR4 | CRD_FL_c38346_g2_i1 | jasmonic acid carboxyl methyltransferase | CDS | F: AGGGGAAGGAGAGACTAGCT  R: TTGGGTCCTGATGAGCAACC | (CAA)4 | 57 | | 171 | N |
| SSR5 | CRD_FB_c34734_g1_i1 | ARM repeat superfamily protein | 5’ UTR | F: CTCTTTTGATCACTTATTGCTT  R: GACCGGACGGTTCGCATGGAC | (AT)8 | 52 | | 251 | E |
| SSR6 | CRD_LB_c46476_g2_i2 | GDSL-like Lipase/Acylhydrolase superfamily protein | CDS | F: GGAAGATGAGGTTGTGGTGT  R: GGCTTTGGCAAGGGTAGCTA | (AGG)5 | 55 | | 173 | E |
| SSR7 | CRD_FL_c49209_g2_i1 | WD-40 repeat family protein | 3’ UTR | F: TTACCTTCCGTCGCCTCAA  R: AATGGCCTCATCACCCAAAT | (TA)7 | 53 | | 253 | E |
| SSR8 | CRD_FR_c29094_g1_i1 | cytochrome P450 superfamily | 3’ UTR | F: CTTGGTTTGATGATGTTATGAAGTG R: TCAAAGGGAAAAAACAACAAATC | (GT)7 | 52 | | 142 | E |
| SSR9 | CRD_FL_c23698_g1_i1 | - | - | F: GCCCAAGTAAGATTGGATGAAC  R: GGGTTTCTAATATCTTTATGGTTTG | (TA)6 | 54 | | 160 | E |
| SSR10 | CRD_LB_c50221_g1_i1 | - | - | F: GTCCAAATCTCAATTACCGAGGTC  R: ACGTTCTGCCTTAGCACTATGC | (TC)8 | 58 | | 118 | E |
| SSR11 | CRD_LB_c50226_g2_i1 | glycine-rich RNA-binding protein | CDS | F: GCGATGTCAGCAATGGATG  R: ACCATCATTCTCTCTGGCATCAC | (GGT)7 | 55 | | 159 | L |
| SSR12 | CRD_FR_c29919_g1_i2 | proline-rich family protein | CDS | F: CTCCAGTGATGCCACCAATG  R: TCCCTGGAACTGCTGATGG | (GCT)4 | 58 | | 114 | E |
| SSR13 | CRD_LB_c46862_g1_i1 | - | - | F: TGGTGGCGGTGGTTGGATG  R: CGTCTGCCCAGTGCCCTCT | (TC)6 | 58 | | 141 | E |
| SSR14 | CRD_FL_c51717_g3_i3 | - | - | F: AGCTCTGAAGATCTATGGCGGATC  R: TTCAGACGACGATGGCGAG | (TC)6 | 58 | 111 | | E |
| SSR15 | CRD_ML_c84178_g1_i1 | - | - | F: TCACCAGCACCGCTATCATC  R: TGTGGAAGCAGAGGCGAGAT | (GA)8 |  | 157 | | N |
| SSR16 | CRD_FB_c38967_g1_i1 | hypothetical protein | 5’ UTR | F: AATGCACAGCAAAACCTCTGG  R: TCCCACTACATGTGCTGGTG | (AG)7 | 55 | 121 | | E |
| SSR17 | CRD_ML_c87820_g13_i1 | - | - | F: TGGGGTTTCTGTATTTCCGA  R: GCCATGAAAATCGCTGTTGA | (ATC)4 | 55 | 136 | | E |
| SSR18 | CRD_FR_c33636_g1_i2 | hypothetical protein | 5’ UTR | F: GTTTCTGAGTTTCTCTCATCTCTC  R: ACCTCCACGAAATTGCTCAC | (TG)9 | 53 | 126 | | E |
| SSR19 | CRD_FR_c12679_g1_i1 | ubiquitin-conjugating enzyme | 3’ UTR | F: TCAGTTCCGTTTTGTTTCCTTG  R: AACAATATGTCAAGCAAAGCACC | (AT)7 | 53 | 139 | | E |
| SSR20 | CRD_FL_c48774_g1_i1 | hypothetical protein | 5’ UTR | F: TTCGAGCTTGTGATTGTTGAG  R: CCCTAGCTGCAGAAACCATC | (GCTC)3 | 53 | 165 | | E |

E indicates amplified PCR products of the expected size. L and S indicate amplified PCR products larger and smaller than anticipated, respectively. N indicates no amplification.
