# Supplementary material for: Transcriptome Analysis Identifies Candidate Genes Related to Triacylglycerol and Pigment Biosynthesis and Photoperiodic Flowering in the Ornamental and Oil-Producing Plant, Camellia reticulata (Theaceae)
Source: Front Plant Sci. 2016 Feb 23;7:163. doi: 10.3389/fpls.2016.00163 (PMC4763035; doi:10.3389/fpls.2016.00163)
Supplement: Supplementary Table 4 — The top 10 assembled unigenes examined for chimerical assembly errors. [file Table4.DOC]

**Supplementary Table S4 The top 10 assembled unigenes were examined for chimerical assembly errors.**

| **Unigene ID** | **Length (nt)** | **Method** | **Homology ID*** | **Homology length** | **Homology Description** | **Identity** | **Query Coverage** |
| --- | --- | --- | --- | --- | --- | --- | --- |
| CRD_ML_c89441_g4_i3 | 9,880 | BLASTX | EOY24126 | 4,344 AA | Pleckstrin (PH) domain-containing protein isoform 2 [*Theobroma cacao*] | 70% | 94% |
|  |  | megaBLAST | GACF01063530 | 12,175 nt | *Camptotheca acuminata* caa15260_iso6 mRNA sequence | 83% | 94% |
| CRD_FL_c52936_g8_i1 | 8852 | BLASTX | XP_002268896 | 4116 AA | E3 ubiquitin-protein ligase UPL1-like [*Vitis vinifera*] | 78% | 94% |
|  |  | megaBLAST | KA241672 | 9788 AA | *Betula platyphylla* mRNA sequence | 80% | 92% |
| CRD_FL_c52940_g4_i2 | 8827 | BLASTX | XP_002283711 | 3750 nt | E3 ubiquitin-protein ligase UPL2-like [*Vitis vinifera*] | 79% | 95% |
|  |  | megaBLAST | GACF01006232 | 11702 AA | *Camptotheca acuminata* mRNA sequence | 85% | 97% |
| CRD_FL_c51422_g1_i1 | 8239 | BLASTX | XP_003633742 | 6279 AA | LOW QUALITY PROTEIN: auxin transport protein BIG-like [*Vitis vinifera*] | 84% | 97% |
|  |  | megaBLAST | GACF01012454 | 15890 nt | *Camptotheca acuminata* mRNA sequence | 86% | 97% |
| CRD_FL_c52936_g6_i1 | 7776 | BLASTX | XP_002268896 | 4116 AA | E3 ubiquitin-protein ligase UPL1-like [*Vitis vinifera*] | 80% | 95% |
|  |  | megaBLAST | GAKH01099929 | 12175 nt | *Vitis vinifera* transcribed RNA sequence | 82% | 94% |
| CRD_FL_c52959_g2_i5 | 7733 | BLASTX | XP_002277575 | 2394 AA | uncharacterized protein LOC100266406 [*Vitis vinifera*] | 89% | 69% |
|  |  | megaBLAST | GACF01040558 | 7730 nt | *Camptotheca acuminata* mRNA sequence | 81% | 92% |
| CRD_FB_c38277_g2_i1 | 7630 | BLASTX | XP_002264755 | 1170 AA | protein GIGANTEA-like [*Vitis vinifera*] | 85% | 46% |
|  |  | megaBLAST | GBHI01072724 | 3173 nt | *Camellia oleifera* transcribed RNA sequence | 99% | 41% |
| CRD_FL_c48470_g2_i1 | 7630 | BLASTX | XP_002264755 | 2,609 AA | dnaJ homolog subfamily C member 13-like [*Vitis vinifera*] | 88% | 86% |
|  |  | megaBLAST | GAKH01094777 | 8,642 nt | *Vitis vinifera* transcribed RNA sequence | 85% | 83% |
| CRD_ML_c85849_g1_i1 | 7550 | BLASTX | XP_003632762 | 2,347 AA | pre-mRNA-processing-splicing factor 8-like isoform 2 [*Vitis vinifera*] | 98% | 93% |
|  |  | megaBLAST | GAKH01030430 | 7,483 nt | *Vitis vinifera* transcribed RNA sequence | 88% | 93% |
| CRD_FR_c31830_g4_i2 | 7493 | BLASTX | EOY16075 | 2269 AA | Acetyl-CoA carboxylase 1 isoform 1 [*Theobroma cacao*] | 87% | 91% |
|  |  | megaBLAST | GACF01027951 | 6917 nt | *Camptotheca acuminata* mRNA sequence | 88% | 88% |

*In protein level, we used BLASTX tool to search against the NR database in NCBI and extracted the top 10 hit of each sequence for analysis. In cDNA level, we used megaBLAST tool to search against the Transcriptome Shotgun Assembly (TSA) database in NCBI and extracted the top 10 hit of each sequence for analysis. For each unigene, only the homolog sequence with the longest alignment length was showed here.
