# Supplementary material for: Transcriptome Analysis Identifies Candidate Genes Related to Triacylglycerol and Pigment Biosynthesis and Photoperiodic Flowering in the Ornamental and Oil-Producing Plant, Camellia reticulata (Theaceae)
Source: Front Plant Sci. 2016 Feb 23;7:163. doi: 10.3389/fpls.2016.00163 (PMC4763035; doi:10.3389/fpls.2016.00163)
Supplement: Supplementary Table 6 — The 10 most abundant PFAM families/domains for C. reticulata unigenes. [file Table6.DOC]

**Supplementary Table S6 The 10 most abundant PFAM families/domains for *C. reticulata* unigenes.**

| **Pfam name** | **Accession ID** | **Number of hits** |
| --- | --- | --- |
| **Family** |  |  |
| Pentatricopeptide repeat (PPR) | PF01535.15 | 3362 |
| Reverse transcriptase (RVT_2) | PF07727.9 | 503 |
| Reverse transcriptase (RVT_1) | PF00078.22 | 490 |
| Leucine-rich repeat (LRR_4) | PF12799.2 | 472 |
| Ankyrin repeat (Ank_2) | PF12796.2 | 422 |
| Mitochondrial carrier protein (Mito_carr) | PF00153.22 | 372 |
| Leucine-rich repeat (LRRNT_2) | PF08263.7 | 304 |
| Mitochondrial carrier protein (Mito_carr) | PF00153.22 | 372 |
| Gag-polypeptide of LTR copia-type (UBN2_3) | PF14244.1 | 237 |
| AAA | PF00004.24 | 229 |
| Glucuronosyltransferases (UDPGT) | PF00201.13 | 222 |
| **Domain** |  |  |
| Protein kinase domain (Pkinase) | PF00069.20 | 2084 |
| Tyrosine kinase (Pkinase_Tyr) | PF07714.12 | 2002 |
| RNA recognition motif (RRM_1) | PF00076.17 | 829 |
| RNA recognition motif (RRM_6) | PF14259.1 | 726 |
| RNA recognition motif (RRM_5) | PF13893.1 | 550 |
| Zinc-binding in reverse transcriptase (zf-RVT) | PF13966.1 | 459 |
| Myb-like DNA-binding domain (Myb_DNA-binding) | PF00249.26 | 457 |
| EF-hand_5 | PF13202.1 | 393 |
| RING finger domain (zf-C3HC4) | PF00097.20 | 392 |
| RING finger domain (zf-RING_2) | PF13639.1 | 387 |
